# Biosynthesis of Grandione: An Example of Tandem Hetero Diels-Alder/Retro-Claisen Rearrangement Reaction?

**DOI:** 10.3390/molecules23102505

**Published:** 2018-09-30

**Authors:** Ramiro F. Quijano-Quiñones, Carolina S. Castro-Segura, Gonzalo J. Mena-Rejón, Mariana Quesadas-Rojas, David Cáceres-Castillo

**Affiliations:** Chemistry Faculty, Autonomous University of Yucatan, 97069 Mérida, Yucatán, Mexico; A11019512@alumnos.uady.mx (C.S.C.-S.); mrejon@uady.mx (G.J.M.-R.); mariana.quesadas@gmail.com (M.Q.-R.); david.caceres@correo.uady.mx (D.C.-C.)

**Keywords:** icetexane dimer, grandione, tandem reaction, DFT

## Abstract

Mechanistic theoretical studies about the feasibility of the traditional proposed mechanism of formation for icetexane diterpene dimer grandione were assessed using density functional method at the M06-2X/6-31G(d,p) level of theory. Bulk water solvent effects were taken into account implicitly using the polarizable continuum model (SCI-PCM). The results were compared with the selectivity found in the biomimetic synthesis performed by experimental research groups. The relative free energy calculation shows that the one-step H-DA formation mechanism nominated in the literature is not a viable mechanism. We found that an alternative competing Tandem pathway is consistent with the experimental trends. Thus, our results suggested that the compound grandione is formed via a H-DA/retro-Claisen rearrangement and not by the traditional H-DA mechanism proposed early in the experimental studies. The H-DA initial step produce a biecyclic adduct followed by a domino retro-Claisen rearrangement that releases the energy strain of the bicyclic intermediary. Steric issues and hyperconjugation interactions are the mainly factors driving the reaction nature and the selectivity in the formation reaction. Finally, the enzymatic assistance for dimer formation was analyzed in terms of the calculated transition state energy barrier.

## 1. Introduction

The Diels-Alder (DA) and the Hetero Diels-Alder (H-DA) reactions are important ring-forming reactions in organic synthesis that have been postulated as a key steps in more than 400 biosynthetic conversions [1]. 

However, although enzymes that catalyze biotransformations that could involve a Diels-Alder reaction have been identified [2,3,4,5,6,7,8,9], until now it has been difficult to verify the existence of a natural Diels-Alderase enzyme [10,11,12,13]. Recently, results from synthesis of natural products via biomimetic Diels-Alder reaction suggest that in some cases the DA and H-DA can proceed unenzymatically [14]. Therefore the presence of a cyclohexane moiety in natural products with a defined stereochemistry is not unambiguous proof of Diels-Alderase activity since the cycloaddition can occur without catalysis or, as some studies propose, the transformation could proceed via polar/non-polar mechanisms or via stepwise reactions [12,15,16]. From the computational point of view, several theoretical efforts have been realized to give support or discard the H-DA hypothesis in different natural products [17,18,19,20]. In this sense, the computational tools are valuable since they provide additional information on the biogenesis of natural compounds, such as the geometry and energy of the involved intermediary and transition estate structure and the feasibility of biological enzymatic assisted reactions. Therefore, based on the fact that in vivo chemical reactions follow the same principles of *in vitro* transformation [21,22] it is possible to evaluate proposed biogenetic theories using theoretical models and to help establish the reaction mechanisms by which these transformations occurs. 

**Figure 1:**
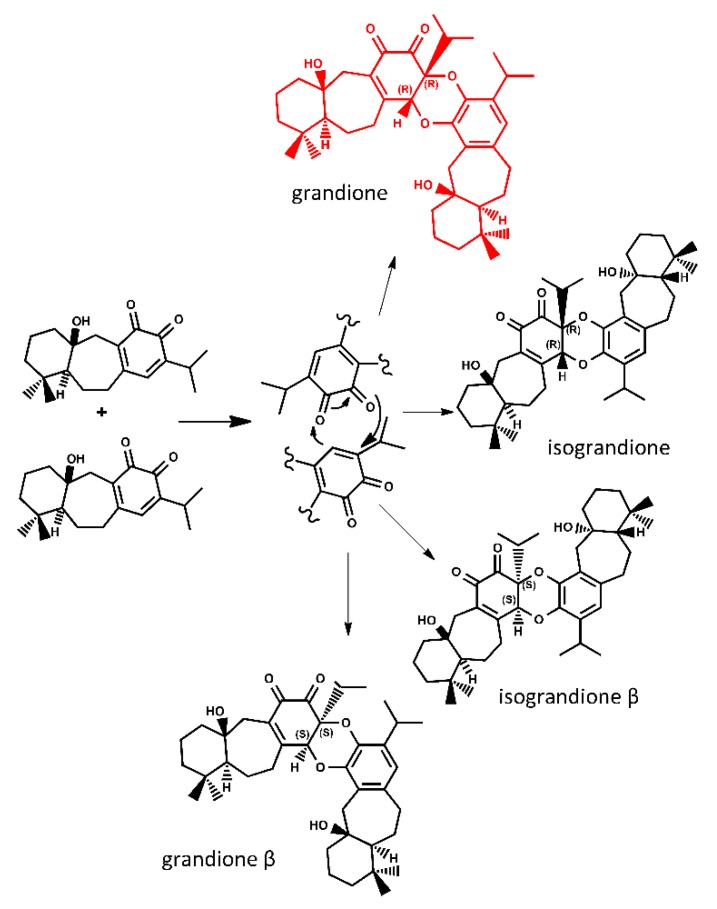
Biosynthetic mechanism for formation of the icetexane dimer grandione proposed in the literature [24,25].

Grandione was the first reported example of an icetexane dimer and was isolated from *Torreya grandis* by Riccio et al. in 1999 [23]. The structure of grandione is based on two rearranged abietane-type monomeric units and it is proposed that the dimer arises as the result of a had-type cycloaddition of demethylsalvicanol quinone, as shown in Figure 1. In 2005, the Takeya group synthesized grandione [24]. Firstly the oxidation of demethylsalvicanol was carried out using several reaction conditions to obtain demethylsalvicanol quinone, later, the quinone was subjected to H-DA conditions in the solid state to obtain only one of the four possible cycloadducts, the compound grandione (Figure 1). In 2008, Majetich and Zou repeated Takeya’s result in water, imitating the natural conditions [25]. Both research groups suggested that a non-enzymatic biomimetic Hetero Diels-Alder reaction of demethylsalvicanol quinone proceeded to produce grandione, efficiently and that this reaction gave primarily one of the four possible isomers.

In order to give insight in to the biogenesis mechanism of grandione, in this work we present a computational study of the proposed formation reaction of this icetexane dimer. The main goal of this paper is to obtain evidence regarding the feasibility of the H-DA reaction and explain the selectivity found in the experimental reports.

## 2. Results and Discussion

The hypothetical biosynthesis of grandione is shown in Figure 1. According to this hypothesis the dimerization of demethylsalvicanol quinone through a H-DA reaction can give four possible cycloadducts. The three hypothetic isomers were named as is shown in Figure 1 along the grandione compound grandione. The optimized geometries of the four adducts are showed in the Supplementary Material (Figure S1). The calculated relative free energies (ΔG_reac_), entropy (ΔS_reac_), and enthalpy contributions (ΔH_act_) in solvent phase (water) for the reactions are summarized in Table 1. From the free energy reaction we can observe that all the reactions were calculated to be exergonic, with a similar energy reaction fluctuating from −20.29 kcal/mol (isograndione) down to −19.07 kcal/mol (grandione). 

For each one of the four possible isomer of the H-DA reaction of demethylsalvicanol quinone, the diene and dienophile may arrange themselves in two different ways (*exo* and *endo*) giving a total of eight possible H-DA transition states. The Cartesian coordinates of the stationary points associated with the reactions studied are listed in the Supplementary Material.

**Figure 2:**
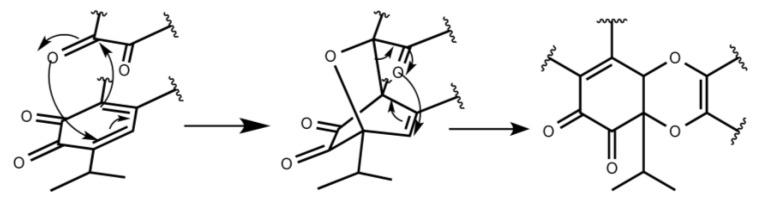
Schematic view of the proposed Tandem mechanism for the formation of isograndione and grandione compounds.

**Figure 3:**
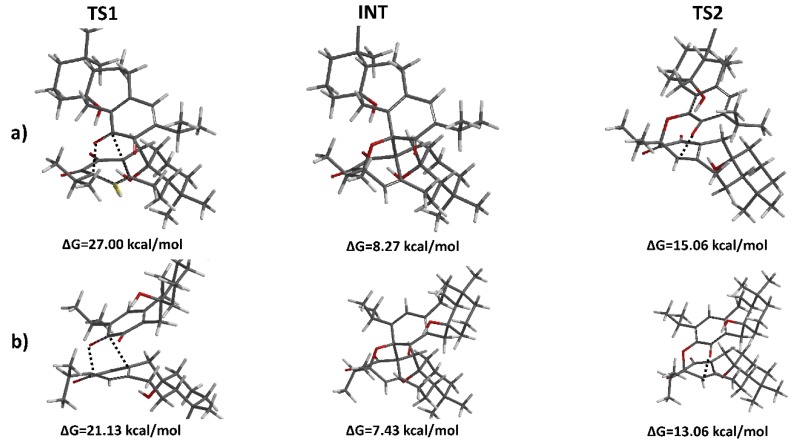
Equilibrium geometries of intermediaries and transition states and their relative energies for the domino process to lead to (**a**) the isograndione and (**b**) grandione molecules.

The activation free energies (ΔG_act_) for the H-DA mechanism were collected in Table 1 along with the enthalpic and entropic contributions. From these results we obtain that ΔG_act_ fluctuates from 33.4 kcal/mol (isograndione β, *endo*) up to 43.9 kcal/mol (isograndione β, *exo*). These results cannot explain the higher selectivity leading to the compound grandione in the biomimetic synthesis performed by the Takeya [14] and Majetic [25] groups. It is noteworthy to mention that the *endo* HAD transition states of grandione and isograndione cannot be localized. All attempts to find one-step pathways in both cases were unsuccessful. The theoretical results, along with the fact that the biosynthesis gave only grandione as product, suggested exploring alternative competing mechanisms that can explain these observations. Domino reactions, involving a combination of H-DA, retro-Claisen and retro-Cope rearrangement, have been proposed to lead to H-DA type cycloadducts [26,27,28,29,30,31,32,33,34,35] and have been described recently by Jasinski [36]. These findings prompted us to consider a possible tandem reaction pathway. An initial [4+2] H-DA reaction, followed by a domino retro-Claisen rearrangement, as shown in Figure 2, would give arise to a H-DA-type adduct. In order to prove this hypothesis, we engaged in a search for the two-step mechanism in all compounds. Surprisingly, only grandione and isograndione present the proposed tandem pathway. The results are collected in Table 2 and the stationary points of both reaction pathways in Figure 3. The Cartesian coordinates of the stationary points are recorded in the Supplementary Material. The results showed that the relative Gibbs energy (ΔG(INT)) of both reaction intermediates is similar and positive, thus the rate limiting step of the tandem mechanism is the first step in both compounds (21 kcal/mol for grandione and 27 kcal/mol for isograndione) since it presents the highest relative Gibbs energy on the reaction coordinates. It is interesting to note that the activation energy of the tandem reaction is significantly lower than the activation energy in the one-step cases. In order to understand the reason behind these trends we applied EDA calculations to the grandione and isograndione adducts. The results, collected in Table 3, show that *∆E_str_ (ζ)* in one-step case is lower than in the case of the H-DA of the tandem process. Thus, it is the stabilizing interaction *∆E_int_ (ζ)* that causes the lower energy barrier in the cascade pathway. In addition, the ΔG_act_(TS1) in grandione is nearly 6 kcal/mol lower in energy than ΔG_act_(TS1) of isograndione and thus we can conclude that the isograndione, isograndione β, and grandione β cycloadducts are either minor products or not detected. The results explain satisfactorily the experimental trends and suggest an alternative rationalization of the mechanism proposed by Takeya et al. [14].

In addition, the Takeya group [14] demonstrated that the reaction of dimethylsalvicanol quinone leading to grandione can proceed non-enzymatically. Is the barrier calculated in this work small enough for the tandem reaction can occur without enzymatic intervention? In order to address this question we need taking to account that most of the chemical reactions occurring in the metabolic process of living cells have an activation barrier of 10–20 kcal/mol [37]. The predicted barrier for the determining step of the uncatalyzed reaction cascade producing grandione is around 21 kcal/mol. This barrier is near the limit of attainability for a biological reaction, however, due to the limitations of the theoretical DFT methods we cannot conclude with certainty if the reaction would require or not enzymatic intervention in a biological environment. Nevertheless, our results suggested that the reaction is energetically feasible under the experimental conditions used by Majetic et al. in agreement with the experimental observations [14,25].

For all TS’s of the Diels Alder reactions the global electron density transfer (GEDT) was calculated [38]. The GEDT was computed by sharing the natural charges obtained from an NBO analysis [39,40,41,42,43,44,45]. The results are shown in Table S1 in the supplementary materials. The GEDT calculated fluctuates from 0.22 e (TS1 isograndione) up to 0.29 e (TS1 grandione). According to the scale introduced by Domingo et al. [46], it can be concluded that these reactions have a polar character. The electronic flux indicates that the one-step H-DA reaction that leads directly to grandione presents an inverse electronic demand, meanwhile the H-DA for the first step from the tandem process presents normal electronic demand, moreover the activation energy has been inversely related to the GEDT [46]. Analyzing Table S1, we can see that our findings support it. Furthermore, from these results, the difference in the activation free energy between TS1 in grandione and isograndione can be explained. The computed GEDT values at TS1 are are 0.27 e at grandione, 0.22 e at isograndione, and the activation free energies values are 21.13 kcal/mol and 27.00 kcal/mol, respectively. Thus, grandione have the lowest activation free energy since it presents a more polar character. To obtain a better understanding on the factors that control the nature and the selectivity of the formation reaction of grandione, in the next section we focus on analyzing in detail its domino formation reactions.

### Tandem Reaction Analysis of Gradione

**Figure 4:**
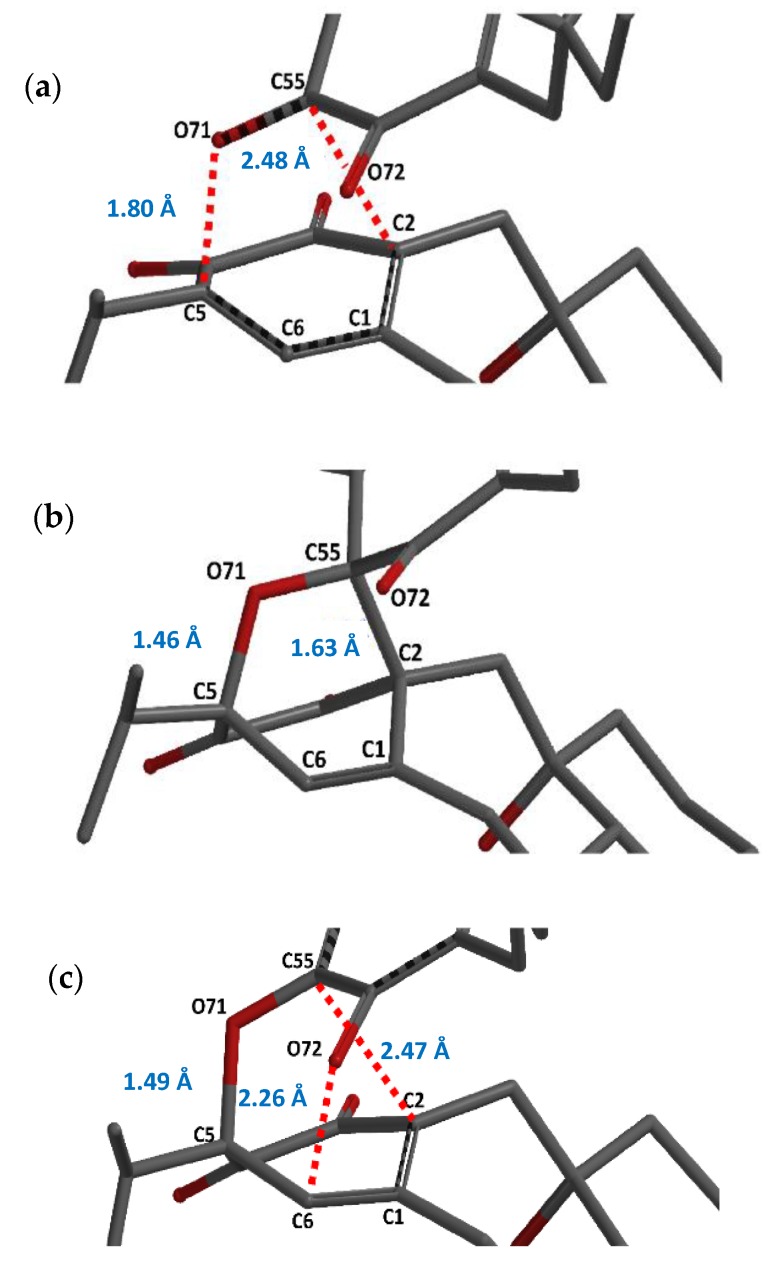
Structure of the TS1 (**a**) and the intermediate (**b**) and TS2 (**c**) transition states for the icetexane dimer grandione showing the main interatomic distances.

**Figure 5:**
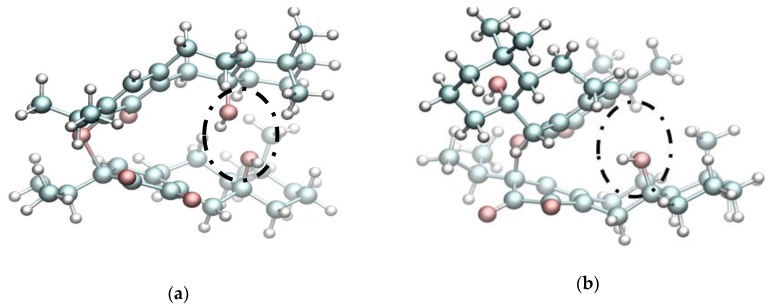
Equilibrium geometries of the endo transition state of grandione β (**a**) and isograndione β (**b**) showing an emergent steric repulsion due to the hydroxyl moiety orientation.

As was discussed in the results of the previous section, grandione is formed by a H-DA/retro-rearrangement Claisen. The first stage is an asymmetric Hetero Diels-Alder reaction where the carbonyl moiety in C55 on the orthoquinone system acts as the dienophile reacting with the conjugated diene as is showed in Figure 4a. The C5-O71 bond formation is characterized by a length distance of 1.8 Å, in contrast with the length distance of the C55-C2 which is 2.48 Å. Thus, the calculation predicted asynchronous transition structures in the first stage. In addition, as we mentioned early, the GEDT shows that the H-DA reactions present normal electronic demand with a value of 0.27 e. Why this reaction take place? In order to answer this question, NBO analysis was carried in the early structures in the IRC path going to reactants toward TS1 in grandione and isograndione where the length distance of the most formed bond is around 2.18 Å. We have considered all possible interactions between the two fragments. In the early stage of the grandione dimer formation, the complex is mainly stabilized due to hyperconjugation interaction that results from the $\pi_{C2-C1}\to \pi_{C55-O71}^{*}$. The second order NBO analysis of these interactions establishes values around of 6.78 kcal/mol for the stabilization energy. Similar results were found in the isograndione case, where the hyperconjugation interaction is around 6.84 kcal/mol. It is remarkable that in the case of grandione β and isograndione β in *endo* approximation (in the *exo* one the tandem reaction is not possible due to geometric issues) the reactant fragments approach one another with their hydroxyl groups oriented towards each other, so the resulting steric repulsion impedes the formation of the hyperconjugation interaction leading the reaction trough a one-step way (Figure 5). It can be deduced from our results that, the steric bulk and hyperconjugation interaction are guiding the first reaction of the tandem process.

The H-DA initial reaction produces a bicyclic system intermediate with a bicyclo[2.2.2]octane skeleton (Figure 4b) with an energy difference of 7.4 kcal/mol in comparison with the isolated precursors. The O71-C5 bond distance is 1.46 Å meanwhile the C55-C2 bond length is 1.63 Å showing that the O71-C5 bond is more formed than the C55-C2 bond. In this stage, the bicyclic intermediate has enough strain energy to experience a retro-Claisen rearrangements giving an unsaturated oxa- retro-Claisen product, grandione. Thus, the strain-release on going from the bridged compound to final product serves as a driving force for the retro-rearrangement. Other rearrangements driven by strain inherent to bicyclic systems have been reported [47].

The transition state of the retro-Claisen rearrangement (TS2) (Figure 4c) has an activation free energy of 13.06 kcal/mol, 2 kcal/mol lower than in the isograndione case. In the one hand, the TS2 the C55-C2 distance increases from 1.63 Å (INT) up to 2.47 Å and the C72-C6 decreases from 2.96 Å (value in the INT structure) down to 2.26 Å showing that a new bond is formed while the other is broken. On the other hand, the C5-O71 bond length slightly increases from 1.46 Å up to 1.49 Å. Finally, the metabolite grandione is formed, as we have previously discussed, by reducing the strain in the bicyclic system by opening the four-membered ring.

## 3. Materials and Methods 

### Computational Details

The full geometrical optimization of all structures (reactants, transition state structures, and products) were carried out using DFT methods at the M06-2X/6-31G(d,p) level of theory. In the case of the reactants and products, an initial conformer search was performed using a Monte Carlo method with a starting temperature of 5000 K as it is implemented in the Spartan16 software [48]. Next, the most stable conformer was full optimized without restrictions. The water solvent effects was evaluated by using the conductor-like polarizable continuum model (CPCM) with UAKS radii [49,50,51]. The meta-GGA M06-2X [52] functional was applied since performs better with π→σ transformations in reactions such as Diels-Alder and [3+2] cycloadditions reactions [53,54,55,56]. In addition, the M06-2X functional has been shown to yield more accurate barrier eighth energies for cycloaddition reactions although, it underestimates the value of the activation energy [57]. In a previous work, the M06-2X functional has proven to be successful in the study of the terminal biogenesis of triterpene dimers [20].

Normal vibrational mode analysis were performed to characterize the stationary points at the same computational level. Zero-point vibrational energy corrections were applied without scaling for all stationary points. The Schlegel Synchronous Transit-Guided Quasi-Newton (QST2) and the QST3 methods were used to locate each TS between two minima [58]. Additionally, the transition states have been verified by intrinsic reaction coordinate (IRC) [59] calculation. All the calculations were performed at 298 K and 1 atm.

The free energies of activation (ΔG_act_) for the reactions were computed by subtracting the sum of free energies of isolated reactants from the free energy of the optimized. The free energies of reaction (ΔG_react_) were calculated from the differences between the free energies of products and reactants. All calculations were carried out with the Gaussian 09 suite [60].

Where necessary, a natural bond orbital analysis (NBO) were performed. All the possible interactions between filled donor NBO (*i*) and almost empty acceptor NBO (*j*) was calculated. The stabilization energy *E*(2) associated with the electron delocalization was estimate using a second order perturbation as:

$$\;E\left(2\right)=q_{i}\frac{{\left(F_{i,j}\right)}^{2}}{E_{j}-E_{i}}\;$$

where *q_i_* is the orbital occupancy, *E_i_* and *E_j_* are the diagonal elements (orbital energies) and *F*(*i,j*) is the off-diagonal NBO Fock matrix element. The NBO analysis were performed with the version included in Gaussian 09 [41,42,43,44,45]. Finally, the NBO visualization was achieved with the software Multiwfn 3.5 [35]. In order to rationalize the activation energy of the reaction in term of the reactants, an Energy Decomposition Analysis (EDA) was computed [61]. In this approach the energy barrier ∆*E* (*ζ*) is partitioned in the energy strain ∆*E_str_* (*ζ*) associated with the geometric deformation of reactants during the chemical reaction, plus the interaction energy ∆*E_int_* (*ζ*) between the deformed reactants which depends on their electronic structure and on their mutual orientation as they approach each other:

$$\;\Delta E\left(\zeta \right)=\Delta E_{str}\left(\zeta \right)+\Delta E_{int}\left(\zeta \right)\;$$

## 4. Conclusions

In summary, we have analyzed the feasibility of the Hetero Diels-Alder cycloaddition in the biosynthetic mechanism of the icetexane dimer grandione using the DFT method at the M06-2X/6-31G(d,p) computational level. We establish that the one-step H-DA transformation mechanism proposed in the literature cannot account for the experimental results. We found that an alternative competing mechanism could explain this observation. Our results suggested that grandione is formed through a tandem reaction where the initial step is a H-DA cycloaddition to produce a bicyclic adduct followed by a domino retro-Claisen rearrangement. Steric impediments prevent grandione β and isograndione β from being formed via the domino reaction. Our analysis shows that, in the first stage, steric bulk guides the first reaction due to the hydroxyl group orientations and hyperconjugation interaction orbitals, while the strain-release is the driving force in the second process. Thus, steric issues and hyperconjugation interaction are the determining factors driving the reaction nature and the selectivity in the reaction of formation of the compound grandione. Finally, the activation free energy in the mechanism formation of grandione, did not show decisive information to relate this transformation with an enzymatic process, in consequence it is necessary to increase the theoretical and experimental evidence in order to clarify the biosynthesis of grandione.

## Figures and Tables

**Table 1:** Relative enthalpies, entropies, and Gibbs energies (kcal/mol) for the stationary points involved in the DA reactions at 298.15 K and 1 atm in water.

Molecule				*Exo*			*Endo*		
	ΔG_reac_	ΔH_reac_	TΔS_reac_	ΔG_act_	ΔH_act_	TΔS_act_	ΔG_act_	ΔH_act_	TΔS_act_
Grandione	−19.07	−36.31	−17.24	37.31	20.85	−16.45	-	-	-
Grandione β	−19.52	−38.51	−18.99	40.98	22.43	−18.56	44.26	25.79	−18.47
Isograndione	−20.29	−38.54	−18.24	37.74	22.13	−15.61	-	-	-
Isograndione β	−20.26	−38.84	−18.58	43.94	25.95	−17.99	33.43	15.80	−17.63

**Table 2:** Relative energy Gibbs reactions (kcal/mol) for the stationary points involved in the Tandem reactions at 298.15 K and 1 atm in water.

Molecule	ΔG_act_(TS1)	ΔG(INT)	ΔG_act_(TS2)	ΔG_reac_
Grandione	21.13	7.43	13.06	−19.07
Isograndione	27.00	8.27	15.06	−20.29

**Table 3:** Deformation and interaction energy (kcal/mol) at the TS in grandione and isograndione.

Molecule	*Exo*		TS1	
	∆*E_str_ (ζ)*	∆*E_int_ (ζ)*	∆*E_str_ (ζ)*	∆*E_int_ (ζ)*
Grandione	23.42	−2.73	27.68	−23.66
Isograndione	23.90	−1.84	35.61	−27.66

## Supplementary Materials

The following are available online. Figure S1: M06-2X/6-31G(d,p) optimized geometries of grandione (a), isograndione (b), grandione β (c) and isograndione β (d); Table S1: Global electron density transfer (GEDT, in e) for all H-DA transition states; Cartesian coordinates of all the stationary points for transition states, products and intermediaries included in this study at M06_2x/6-31G(d,p) level of theory.

## References

[1] Oikawa H., Mander A., Lui H.W. (2010). Diels-Alderases. Comprehensive Natural Products II Chemistry and Biology.

[2] Auclair K., Sutherland A., Kennedy J., Witter D.J., Van den Heever J.P., Hutchinson C.R., Vederas J.C.J. (2000). Lovastatin Nonaketide Synthase catalyzes an intramolecular Diels-Alder reaction of a substrate analogue. J. Am. Chem. Soc..

[3] Ose T., Watanabe K., Mie T., Honma M., Watanabe H., Yao M., Oikawa H., Tanaka I. (2003). Insight into a natural Diels-Alder reaction from the structure of macrophomate synthase. Nature.

[4] Ma S.M., Li J.W.H., Choi J.W., Zhou H., Lee K.K.M., Moorthie V.A., Xie X., Kealey J.T., Da Silva N.A., Vederas J.C. (2009). Complete reconstitution of a highly reducing iterative polyketide synthase. Science.

[5] Kim R.R., Illarionov B., Joshi M., Cushman M., Lee C.Y., Eisenreich W., Fischer M., Bacher A. (2010). Mechanistic insights on Riboflavin Synthase inspired by selective binding of the 6,7-dimethyl-8-ribityllumazine exomethylene anion A. J. Am. Chem. Soc..

[6] Kim H.J., Ruszczycky M.W., Choi S., Liu Y., Liu H. (2011). Enzyme-catalysed [4+2] cycloaddition is a key step in the biosynthesis of Spinosyn A. Nature.

[7] Fage C.D., Isiorho E.A., Liu Y., Wagner D.T., Liu H., Keatinge-Clay A.T. (2015). The structure of SpnF, a standalone enzyme that catalyzes [4+2] cycloaddition. Nat. Chem. Biol..

[8] Hashimoto T., Hashimoto J., Teruya K., Hirano T., Shin-ya K., Ikeda H., Liu H., Nishiyama M., Kuzuyama T. (2015). Biosynthesis of Versipelostatin: Identification of an enzyme-catalyzed [4+2]-cycloaddition required for macrocyclization of spirotetronate-containing polyketides. J. Am. Chem. Soc..

[9] Tian Z., Sun P., Yan Y., Wu Z., Zheng Q., Zhou S., Zhang H., Yu F., Jia X., Chen D. (2015). An enzymatic [4+2] cyclization cascade creates the pentacyclic core of pyrroindomycins. Nat. Chem. Biol..

[10] Townsend C.A. (2011). A “Diels-Alderase” at Last. ChemBioChem.

[11] Kelly W.L. (2008). Intramolecular cyclizations of polyketide biosynthesis: Mining for a “Diels-Alderase”?. Org. Biomol. Chem..

[12] Kim H.J., Ruszczycky M.W., Liu H.W. (2012). Current developments and challenges in the search for a naturally selected Diels-Alderase. Curr. Opin. Chem. Biol..

[13] Byrne M.J., Lees N.R., Han L.C., van der Kamp M.W., Mulholland A.J., Stach J.E.M., Willis C.L., Race P.R. (2016). The catalytic mechanism of a natural Diels-Alderase revealed in molecular detail. J. Am. Chem. Soc..

[14] Oikawa H., Tokiwano T. (2004). Enzymatic catalysis of the Diels-Alder reaction in the biosynthesis of natural products. Nat. Prod. Rep..

[15] Singleton D.A., Schulmeier B.E., Hang C., Thomas A.A., Leung S.-W., Merrigan S.R. (2001). Isotope effects and the distinction between synchronous, asynchronous, and stepwise Diels-Alder reactions. Tetrahedron.

[16] Jasiński R. (2016). A reexamination of the molecular mechanism of the Diels-Alder reaction between tetrafluoroethene and cyclopentadiene. React. Kinet. Mech. Cat..

[17] Chen N., Zhang F., Wu R., Hess A.B. (2018). Biosynthesis of Spinosyn A: A [4+2] or [6+4] cycloaddition?. ACS Catal..

[18] Maiga-Wandiam B., Corbu A., Massiot G., Sautel F., Yu P., Lin B., Houk K.N., Cossy J. (2018). Intramolecular Diels-Alder approaches to the decalin core of Verongidolide: The origin of the exo-selectivity, a DFT analysis. J. Org. Chem..

[19] Kokkonda P., Brown K.R., Seguin T.J., Wheeler S.E., Vaddypally S., Zdilla M.J., Andrade R.B. (2015). Biomimetic total syntheses of (−)-Leucoridines A and C through the dimerization of (−)-Dihydrovalparicine. Angew. Chem..

[20] Quesadas-Rojas M., Mena-Rejón G.J., Cáceres-Castillo D., Cuevas G., Quijano-Quiñones R.F. (2016). Biogenesis of triterpene dimers from orthoquinones related to quinonemethides: Theoretical study on the reaction mechanism. Molecules.

[21] Herrera A.L. (1942). A new theory of the origin and nature of life. Science.

[22] Negrón-Mendoza A., Alfonso L. (1994). Herrera: A Mexican pioneer in the study of chemical evolution. J. Biol. Phys..

[23] Galii B., Gasparrini F., Lanzotti V., Misiti D., Riccio R., Villani C., He G., Ma Z., Yin W. (1999). Grandione, a new heptacyclic dimeric diterpene from *Torreya grandis* Fort. Tetrahedron.

[24] Aoyagi Y., Takahashi Y., Satake Y., Fukaya H., Takeya K., Aiyama R., Matsuzaki T., Hashimoto S., Shiina T., Kurihara T. (2005). Biomimetic synthesis of grandione from Demethylsalvicanol via hetero-Diels-Alder type dimerization and structure revision of Grandione. Tetrahedron Lett..

[25] Majetich G., Zou G. (2008). Total Synthesis of (−)-Barbatusol, (+)-Demethylsalvicanol, (−)-Brussonol, and (+)-Grandione. Org. Lett..

[26] Oppolzer W., Francotte E., Bättig K. (1981). Total synthesis of (±)-Lysergic acid by an intramolecular imino-Diels-Alder reaction. Preliminary communication. Helv. Chim. Acta.

[27] Ismail Z.M., Hoffmann H.M.R. (1982). New dihydropyrans: Lewis acid catalyzed cycloadditions of α,β-unsaturated acyl cyanides to simple, unactivated olefins and dienes: A readily accessible route to derivatives of rose oxide. Angew. Chem. Int. Ed..

[28] Boeckman R.K., Flann C.F.M., Poss K.M. (1985). Synthetic and mechanistic studies of the retro-Claisen rearrangement: An example of cation acceleration of a [3,3]-sigmatropic rearrangement. J. Am. Chem. Soc..

[29] Hanessian S., Compain P. (2002). Lewis acid promoted cyclocondensations of α-ketophosphonoenoates with dienes—From Diels-Alder to hetero Diels-Alder reactions. Tetrahedron.

[30] Wu H.J., Chern J.-H. (1997). Synthesis of 4-oxo- and 4-*anti*-formyl-8,10,12,13-tetraoxapentacyclo-[5.5.1.0^2,6^.0^3,11^.0^5,9^]tridecanes. Tetrahedron.

[31] Arimori S., Kouno T., Okauchi T., Minami T. (2002). The first synthesis of Phosphonoacrolein. Application to Diels-Alder reaction as heterodiene. J. Org. Chem..

[32] Çelebi-Ölçüm N., Ess D.H., Aviyente V., Houk K.N. (2007). Lewis acid catalysis alters the shapes and products of bis-pericyclic Diels-Alder transition states. J. Am. Chem. Soc..

[33] Desimoni G., Faita G., Toscanini M., Boiocchi M. (2007). Peri- and enantioselectivity of thermal, scandium-, and [Pybox/Scandium]-catalyzed Diels-Alder and hetero-Diels-Alder reactions of methyl (E)-2-oxo-4-aryl-butenoates with cyclopentadiene. Chem.-Eur. J..

[34] Zou Y., Wang Q., Goeke A. (2008). Organocatalytic multicomponent α-methylenation/Diels-Alder reactions: A versatile route to substituted cyclohexenecarbaldehyde derivatives. Chem.-Eur. J..

[35] Lu T., Chen F.W. (2012). Multiwfn: A multifunctional wavefunction analyzer. J. Comp. Chem..

[36] Jasiński R. (2017). One-step versus two-step mechanism of Diels-Alder reaction of 1-chloro-1-nitroethene with cyclopentadiene and furan. J. Mol. Graph. Model..

[37] Wang S.C., Tantillo D.J. (2008). Theoretical studies on synthetic and biosynthetic oxidopyrylium-alkene cycloadditions: Pericyclic pathways to Intricarene. J. Org. Chem..

[38] Domingo L.R. (2014). A new C–C bond formation model based on the quantum chemical topology of electron density. RSC Adv..

[39] Carpenter J.E., Weinhold F. (1988). Analysis of the geometry of the hydroxymethyl radical by the “different hybrids for different spins” natural bond orbital procedure. J. Mol. Struct..

[40] Foster J.P., Weinhold F. (1980). Natural hybrid orbitals. J. Am. Chem. Soc..

[41] Carpenter J.E. (1987). Extension of Lewis Structure Concepts to Open-Shell and Excited-State Molecular Species. Ph.D. Thesis.

[42] Reed A.E., Weinhold F. (1983). Natural bond orbital analysis of near-Hartree-Fock water dimer. J. Chem. Phys..

[43] Reed A.E., Weinhold F. (1985). Natural localized molecular orbitals. J. Chem. Phys..

[44] Reed A.E., Weinstock R.B., Weinhold F. (1985). Natural population analysis. J. Chem. Phys..

[45] Reed A.E., Curtiss L.A., Weinhold F. (1988). Intermolecular interactions from a natural bond orbital, donor-acceptor viewpoint. Chem. Rev..

[46] Domingo L.R., Sáez J.A. (2009). Understanding the mechanism of polar Diels-Alder reactions. Org. Biomol. Chem..

[47] Neier R., Banach E. (2016). Applications of Tandem Diels-Alder/sigmatropic rearrangement reactions to natural product synthesis. Curr. Org. Chem..

[48] (2017). Spartan016.

[49] Klamt A., Schüürmann G. (1993). COSMO: A new approach to dielectric screening in solvents with explicit expressions for the screening energy and its gradient. J. Chem. Soc. Perkin Trans. 2.

[50] Andzelm J., Kölmel C., Klamt A. (1995). Incorporation of solvent effects into density functional calculations of molecular energies and geometries. J. Chem. Phys..

[51] Barone V., Cossi M. (1998). Quantum calculation of molecular energies and energy gradients in solution by a conductor solvent model. J. Phys. Chem. A.

[52] Zhao Y., Truhlar D.G. (2008). Density functionals with broad applicability in chemistry. Acc. Chem. Res..

[53] Pieniazek S., Clemente F., Houk K. (2008). Sources of error in DFT computations of C–C bond formation thermochemistries: π→σ transformations and error cancellation by DFT methods. Angew. Chem. Int. Ed..

[54] Hohenstein E.G., Chill S.T., Sherrill C.D. (2008). Assessment of the performance of the M05−2X and M06−2X exchange-correlation functionals for noncovalent interactions in biomolecules. J. Chem. Theory Comput..

[55] Linder M., Brinck T. (2012). Stepwise Diels-Alder: More than just an oddity? A computational mechanistic study. J. Org. Chem..

[56] Tajabadi J., Bakavoli M., Gholizadeh M., Eshghi H. (2016). A mechanistic insight into the effect of piperidine as an organocatalyst on the [3+2] cycloaddition reaction of benzalacetone with phenyl azide from a computational study. Org. Biomol. Chem..

[57] Lan Y., Zou L., Cao Y., Houk K.N. (2012). Computational methods to calculate accurate activation and reaction energies of 1,3-dipolar cycloadditions of 24 1,3-Dipoles. J. Phys. Chem. A.

[58] Peng C., Ayala P.Y., Schlegel H.B., Frisch M.J. (1996). Using redundant internal coordinates to optimize equilibrium geometries and transition states. J. Comput. Chem..

[59] Fukui K. (1981). The path of chemical reactions-the IRC approach. Acc. Chem. Res..

[60] Frisch M.J., Trucks G.W., Schlegel H.B., Scuseria G.E., Robb M.A., Cheeseman J.R., Scalmani G., Barone V., Mennucci B., Petersson G.A. (2009). Gaussian 09, Revision A.1.

[61] Bickelhaupt M.F., Houk K.N. (2017). Analyzing reaction rates with the distortion/interaction-activation strain model. Angew. Chem. Int. Ed..

