# Supplementary material for: Biosynthesis of Grandione: An Example of Tandem Hetero Diels-Alder/Retro-Claisen Rearrangement Reaction?
Source: Molecules. 2018 Sep 30;23(10):2505. doi: 10.3390/molecules23102505 (PMC6222908; doi:10.3390/molecules23102505)
Supplement: Supplementary file 1 [file molecules-23-02505-s001.pdf]

Suplementary Material

List of Contents

	Content	Pages
Figure S1	M06-2X/6-31G(d,p) optimized geometries of: grandione, isograndiene, grandione β, isograndione β.	2
	Global electron density transfer (GEDT, in e) for all HDA transition states	2
Table S1	Cartesian coordinates of all the stationary points for transition states, products and intermediaries included in this study at M06_2x/6-31G(d,p) level of theory.	3

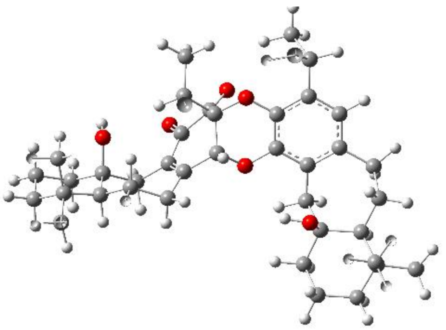

(a)

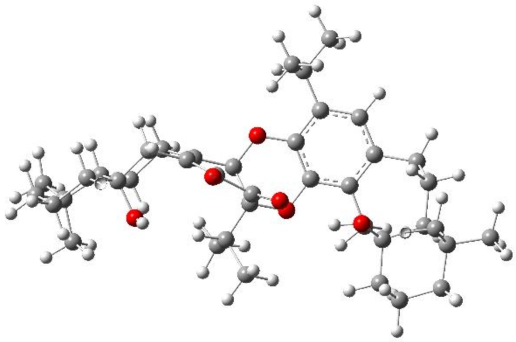

(b)

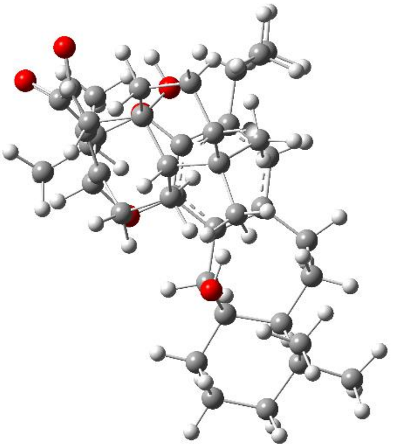

(c)

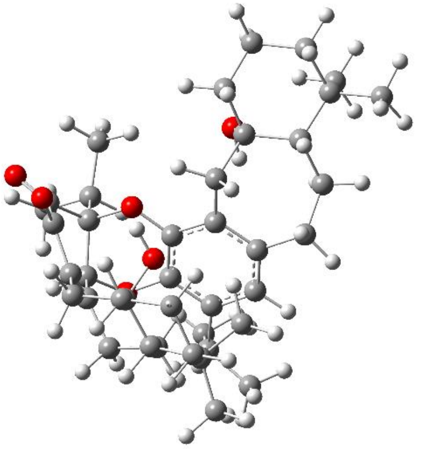

(d)

Figure S1. M06-2X/6-31G(d,p) optimized geometries of grandione (**a**), isograndione (**b**), grandione β (**c**) and isograndione β (**d**).

Table S1. Global electron density transfer (GEDT, in e) for all HDA transition states.

Molecule	GEDT		
	Exo	*Endo*	**TS1**
Grandione	0.286	-	0.266
Grandione β	0.255	0.272	-
Isograndione	0.285	-	0.219
Isograndione β	0.258	0.267	-

Cartesian coordinates of all the stationary points for diterpenes included in this study at M06_2x/6-31G(d,p) level of theory.

Reactive

Demetilsalvicanol quinone

C	0.996500	-0.578800	-0.936100
C	0.646800	0.725800	-0.882900
C	1.604000	1.678300	-0.310400
C	3.048000	1.191300	-0.012400
C	3.344100	-0.252900	-0.141700
C	2.348200	-1.045100	-0.582600
C	-0.720000	1.230600	-1.241700
H	-0.670200	2.323300	-1.289100
H	-1.005400	0.878300	-2.239900
C	-0.040800	-1.612500	-1.283300
H	-0.456500	-1.405100	-2.278600
H	0.430400	-2.597200	-1.338400
C	-1.828200	0.843400	-0.231100
C	-1.182800	-1.649700	-0.244700
H	-0.754500	-1.532700	0.756200
H	-1.623600	-2.648700	-0.286500
C	-2.305500	-0.616700	-0.444100
H	-2.603700	-0.658500	-1.504300
H	2.527900	-2.110200	-0.705700
O	3.877800	2.016100	0.311500
O	1.318800	2.825800	-0.019600
O	-1.311600	0.948400	1.094800
H	-0.956000	1.842800	1.196200
C	-3.589500	-1.000400	0.359900
C	-2.988900	1.830100	-0.413700
H	-3.298200	1.815900	-1.467700
H	-2.615300	2.841200	-0.206700
C	-4.177100	1.492400	0.475700
H	-4.982100	2.217900	0.321500
H	-3.875600	1.565400	1.526800
C	-4.665700	0.084200	0.159500
H	-5.539100	-0.172200	0.771800
H	-4.997300	0.056100	-0.889100
C	-3.314200	-1.192100	1.859000
H	-4.246900	-1.454700	2.370900

H	-2.607200	-2.011000	2.026300
H	-2.900200	-0.292600	2.316300
C	-4.178300	-2.310800	-0.188600
H	-5.166600	-2.479100	0.252900
H	-4.299900	-2.266400	-1.276600
H	-3.565600	-3.183200	0.053600
C	4.733600	-0.707800	0.232300
H	5.426400	0.016200	-0.214700
C	5.072800	-2.101200	-0.289500
H	4.925300	-2.175200	-1.370700
H	6.117300	-2.334700	-0.068200
H	4.455600	-2.865400	0.194800
C	4.915500	-0.641200	1.757100
H	4.710900	0.361800	2.137800
H	4.236600	-1.346200	2.247300
H	5.941200	-0.909100	2.025800

M06-2X/6-31G(d,p) Free Energy = -1003.862032

Adducts

Grandione

C	-1.682600	-0.506400	-0.044200
C	-2.319800	-0.318500	-1.215900
C	-2.049100	0.894400	-2.015700
C	-1.133200	1.981900	-1.405200
C	-0.927500	1.900100	0.103100
C	-0.568200	0.428500	0.360400
C	-3.399600	-1.236500	-1.710700
H	-3.607800	-0.977800	-2.753300
H	-3.054200	-2.276500	-1.698500
C	-2.102900	-1.603300	0.892500
H	-1.999700	-2.577600	0.395700
H	-1.434400	-1.620900	1.757500
C	-4.719300	-1.123500	-0.903100
C	-3.559600	-1.424500	1.372900
H	-3.739200	-0.365200	1.588600
H	-3.652000	-1.958500	2.321700
C	-4.650400	-1.936000	0.416900
H	-4.363500	-2.952800	0.103700
H	-0.292200	0.298800	1.412900
O	-0.641300	2.828700	-2.106900
O	-2.554200	1.118000	-3.097900
O	-4.945300	0.244300	-0.557900
H	-5.083900	0.739100	-1.377400
C	-6.022400	-2.097700	1.150200
C	-5.855000	-1.626800	-1.801300
H	-5.603500	-2.639700	-2.143400
H	-5.891900	-0.992700	-2.696700

C	-7.196400	-1.645200	-1.083500
H	-7.978700	-2.006300	-1.758600
H	-7.469700	-0.624400	-0.793100
C	-7.100600	-2.542700	0.143000
H	-8.064500	-2.594900	0.663900
H	-6.867200	-3.564900	-0.189900
C	-6.469100	-0.808500	1.856200
H	-7.443300	-0.971200	2.330700
H	-5.763500	-0.527500	2.644600
H	-6.548400	0.029400	1.162500
C	-5.920700	-3.211100	2.205500
H	-6.917400	-3.427500	2.605000
H	-5.526300	-4.135300	1.769200
H	-5.288300	-2.933800	3.053100
C	-2.186800	2.390100	0.850600
H	-3.042500	1.843100	0.434000
C	-2.392400	3.888000	0.614700
H	-2.423000	4.135800	-0.450400
H	-3.336300	4.205300	1.064600
H	-1.582400	4.462100	1.073800
C	-2.089900	2.085200	2.347100
H	-2.089000	1.010800	2.554800
H	-1.176800	2.518300	2.767400
H	-2.946800	2.520600	2.867400
C	3.965700	1.433800	-0.550200
C	2.888200	0.559900	-0.710800
C	1.617400	0.996300	-0.327900
C	1.404300	2.293500	0.147900
C	2.471800	3.186100	0.283100
C	3.740800	2.721500	-0.060500
C	3.078500	-0.826400	-1.267000
H	2.097200	-1.249200	-1.499100
H	3.628200	-0.775200	-2.214700
C	5.363700	0.946400	-0.833900
H	5.459100	0.637700	-1.884300
H	6.056700	1.781200	-0.692100
C	3.810400	-1.812900	-0.320700
C	5.791500	-0.219500	0.079100
H	5.419500	-0.028600	1.091300
H	6.883500	-0.208400	0.136000
C	5.349000	-1.622200	-0.371500
H	5.602800	-1.716800	-1.440300
H	4.586200	3.396100	0.055800
O	0.146200	2.718700	0.507200
O	0.561900	0.118200	-0.462700
O	3.385900	-1.590500	1.023100
H	2.418500	-1.602200	1.026700
C	6.163200	-2.748200	0.344600

C	3.419300	-3.233600	-0.751300
H	3.647000	-3.352500	-1.819500
H	2.331100	-3.333600	-0.646200
C	4.145300	-4.302500	0.051900
H	3.840100	-5.299300	-0.282800
H	3.861600	-4.217600	1.106900
C	5.649500	-4.127500	-0.111100
H	6.193900	-4.904100	0.440500
H	5.903400	-4.259700	-1.173500
C	6.095000	-2.643700	1.875700
H	6.665600	-3.464700	2.324700
H	6.539800	-1.705900	2.223500
H	5.068400	-2.683700	2.241300
C	7.641100	-2.670800	-0.073100
H	8.170500	-3.560400	0.285600
H	7.743400	-2.636400	-1.163500
H	8.152200	-1.800600	0.347100
C	2.268100	4.612400	0.758600
C	1.415800	5.414500	-0.234200
H	1.894800	5.448400	-1.217100
H	0.428700	4.959300	-0.351800
H	1.288300	6.442200	0.120200
C	1.693800	4.689900	2.178700
H	0.671700	4.304800	2.208300
H	2.301300	4.109600	2.879200
H	1.679200	5.729800	2.519200
H	3.265600	5.068000	0.784400

M06-2X/6-31G(d,p) Free Energy = -2007.754463

Isograndione

C	-2.519000	1.042500	-0.312600
C	-2.695700	0.629100	0.957400
C	-1.590400	-0.035100	1.678900
C	-0.313600	-0.391300	0.881500
C	-0.435700	-0.337400	-0.636300
C	-1.139300	0.998500	-0.925600
C	-4.025700	0.679900	1.650100
H	-3.859000	0.479600	2.712800
H	-4.462800	1.681800	1.574500
C	-3.687600	1.485000	-1.147000
H	-4.181800	2.343200	-0.671900
H	-3.330400	1.829700	-2.121100
C	-5.032800	-0.365800	1.102600
C	-4.714500	0.348800	-1.349100
H	-4.179400	-0.587400	-1.544700
H	-5.276100	0.585700	-2.256100
C	-5.714500	0.128700	-0.200900

H	-6.135400	1.113000	0.061000
H	-1.168900	1.165800	-2.007800
O	0.699000	-0.702900	1.460100
O	-1.651200	-0.388500	2.839100
O	-4.336100	-1.575000	0.794300
H	-4.011700	-1.952200	1.623900
C	-6.933900	-0.734400	-0.662100
C	-6.068100	-0.627600	2.202200
H	-6.502000	0.334800	2.505300
H	-5.543400	-1.025800	3.080600
C	-7.173500	-1.570400	1.749300
H	-7.890000	-1.726800	2.561800
H	-6.744200	-2.549700	1.508300
C	-7.875100	-0.980500	0.532500
H	-8.695400	-1.629600	0.202900
H	-8.329200	-0.021200	0.822400
C	-6.510900	-2.076400	-1.278500
H	-7.403400	-2.651000	-1.550000
H	-5.930700	-1.923000	-2.194000
H	-5.903800	-2.667700	-0.592100
C	-7.749500	0.038700	-1.711600
H	-8.686400	-0.492700	-1.910600
H	-8.002900	1.042900	-1.354100
H	-7.226100	0.135600	-2.666400
C	-1.182400	-1.582800	-1.163300
H	-2.119800	-1.662200	-0.597100
C	-0.347300	-2.841900	-0.918600
H	-0.062800	-2.950200	0.131900
H	-0.922900	-3.724700	-1.206500
H	0.566700	-2.818100	-1.518500
C	-1.514400	-1.432900	-2.649700
H	-2.232000	-0.628600	-2.838300
H	-0.606400	-1.229300	-3.225200
H	-1.955700	-2.360800	-3.022100
C	3.741000	1.857400	-0.911200
C	2.964900	0.735100	-1.204200
C	1.586200	0.809800	-1.007300
C	0.981400	1.986300	-0.564800
C	1.744000	3.128400	-0.300900
C	3.123900	3.034100	-0.478500
C	3.592100	-0.568900	-1.611500
H	2.815000	-1.233100	-2.000000
H	4.308100	-0.411200	-2.425900
C	5.243300	1.732600	-0.937800
H	5.593600	1.415500	-1.929400
H	5.678800	2.719500	-0.754300
C	4.312800	-1.297200	-0.444800
C	5.767500	0.749700	0.130300

H	5.188900	0.894400	1.048900
H	6.799900	1.030400	0.355900
C	5.749500	-0.742000	-0.246800
H	6.222100	-0.836100	-1.238600
H	3.746300	3.895900	-0.256300
O	-0.371900	2.037200	-0.309100
O	0.843500	-0.329300	-1.228700
O	3.595100	-1.106300	0.770600
H	2.643100	-1.185800	0.598300
C	6.642600	-1.593500	0.712500
C	4.346000	-2.792600	-0.792100
H	4.791100	-2.914300	-1.789500
H	3.308700	-3.145200	-0.860000
C	5.133800	-3.606900	0.223900
H	5.136800	-4.664900	-0.058400
H	4.642900	-3.536200	1.200800
C	6.560300	-3.077700	0.307500
H	7.153000	-3.668100	1.017700
H	7.037300	-3.197900	-0.676800
C	6.247900	-1.433400	2.188300
H	6.891300	-2.066200	2.810300
H	6.385500	-0.399000	2.519300
H	5.206500	-1.705600	2.363200
C	8.115500	-1.177000	0.565800
H	8.753400	-1.891800	1.097300
H	8.422800	-1.170600	-0.486000
H	8.316700	-0.189100	0.988600
C	1.048700	4.367300	0.231500
C	0.698400	4.176500	1.714700
H	1.614100	4.059400	2.304000
H	0.155200	5.046700	2.095900
H	0.077200	3.289100	1.862000
C	1.857200	5.648100	0.033500
H	2.756400	5.651900	0.658100
H	2.164800	5.775100	-1.008400
H	1.255300	6.513900	0.322200
H	0.106300	4.467100	-0.320700

M06-2X/6-31G(d,p) Free Energy = -2007.756403

Grandione β

C	1.665100	-0.065500	-1.776700
C	2.795800	0.648800	-1.600300
C	4.026900	-0.000900	-1.122800
C	4.032700	-1.534400	-1.136300
C	2.701300	-2.143600	-0.715600
C	1.627900	-1.587100	-1.678200
C	2.851100	2.141000	-1.783100

H	3.905200	2.435100	-1.803100
H	2.417400	2.422500	-2.749200
C	0.377300	0.651800	-2.073300
H	0.488900	1.230700	-3.000100
H	-0.424700	-0.072500	-2.229700
C	2.151000	2.935300	-0.654900
C	-0.047800	1.594100	-0.926900
H	0.083600	1.069700	0.028800
H	-1.116600	1.767800	-1.064200
C	0.617900	2.975400	-0.859100
H	0.487800	3.437500	-1.851700
H	1.763000	-2.005700	-2.680000
O	5.013200	-2.145400	-1.481100
O	5.020800	0.600400	-0.760500
O	2.418900	2.303500	0.596600
H	3.379800	2.225100	0.685800
C	-0.135200	3.920200	0.133400
C	2.724700	4.358400	-0.649800
H	2.602200	4.788700	-1.653000
H	3.804100	4.294000	-0.461900
C	2.044800	5.249100	0.381800
H	2.471000	6.256900	0.346700
H	2.242300	4.853600	1.384400
C	0.543500	5.303300	0.118800
H	0.046500	5.947400	0.855000
H	0.375300	5.762500	-0.866700
C	-0.184200	3.379100	1.570900
H	-0.783000	4.056600	2.190300
H	-0.661200	2.393100	1.609900
H	0.808900	3.285100	2.010000
C	-1.585500	4.125100	-0.337900
H	-2.034300	4.959300	0.212100
H	-1.628900	4.364400	-1.406500
H	-2.212100	3.246200	-0.156200
C	2.673900	-3.674000	-0.582300
H	1.683600	-3.908300	-0.172200
C	3.718400	-4.163900	0.424700
H	3.645700	-3.610900	1.365100
H	3.549400	-5.222400	0.637900
H	4.730000	-4.052100	0.028700
C	2.804400	-4.387500	-1.929600
H	1.954100	-4.180400	-2.584700
H	3.724900	-4.097800	-2.442600
H	2.834300	-5.467700	-1.765100
C	-1.490200	-1.486000	1.854000
C	-1.222600	-1.830800	0.522000
C	0.104000	-1.778300	0.080300
C	1.153600	-1.476000	0.959800

C	0.890200	-1.117700	2.283300
C	-0.442400	-1.112000	2.695200
C	-2.322700	-2.279600	-0.410500
H	-1.866800	-2.626600	-1.340500
H	-2.843600	-3.139000	0.029900
C	-2.907200	-1.507000	2.370100
H	-3.321400	-2.521700	2.288700
H	-2.885100	-1.266900	3.437000
C	-3.370200	-1.195800	-0.779400
C	-3.850400	-0.523100	1.658600
H	-3.324600	0.430200	1.512100
H	-4.674300	-0.304500	2.343100
C	-4.447000	-1.010800	0.328300
H	-4.852400	-2.020200	0.506600
H	-0.672000	-0.834400	3.721300
O	2.468500	-1.533500	0.557300
O	0.368900	-2.068000	-1.240000
O	-2.691200	0.028900	-1.050300
H	-2.113600	0.216400	-0.296500
C	-5.671600	-0.138300	-0.102400
C	-4.011200	-1.617700	-2.103600
H	-4.411400	-2.632900	-1.980700
H	-3.217300	-1.668600	-2.857000
C	-5.127600	-0.681000	-2.543100
H	-5.556800	-1.029600	-3.488100
H	-4.716800	0.317900	-2.728000
C	-6.200400	-0.625000	-1.464200
H	-7.028700	0.025600	-1.771300
H	-6.622600	-1.632700	-1.335400
C	-5.333000	1.358700	-0.182600
H	-6.219500	1.917100	-0.504000
H	-5.038400	1.748400	0.797300
H	-4.519000	1.556900	-0.881500
C	-6.819600	-0.314600	0.905200
H	-7.727600	0.155500	0.512100
H	-7.037200	-1.374900	1.074800
H	-6.608800	0.151200	1.871500
C	2.011800	-0.750800	3.237300
C	1.908900	0.723300	3.646200
H	1.938300	1.371600	2.766000
H	0.972200	0.912600	4.182400
H	2.736300	0.992300	4.310300
C	2.019400	-1.667600	4.465500
H	1.103700	-1.545900	5.053000
H	2.098800	-2.718800	4.174000
H	2.866800	-1.423700	5.113000
H	2.958300	-0.889300	2.707400

M06-2X/6-31G(d,p) Free Energy = -2007.755171

Isograndione β

C	2.797100	1.327900	-0.256700
C	2.797100	0.989700	-1.561800
C	1.980400	1.736900	-2.533600
C	1.306500	3.018500	-2.021900
C	0.809500	2.889400	-0.588500
C	2.037200	2.538000	0.283000
C	3.520400	-0.221700	-2.080700
H	3.503800	-0.176900	-3.174000
H	4.572300	-0.207600	-1.774200
C	3.529300	0.461400	0.733400
H	4.580500	0.378000	0.426900
H	3.518300	0.942400	1.713600
C	2.870600	-1.560300	-1.637700
C	2.917200	-0.953900	0.854000
H	1.827700	-0.875900	0.851400
H	3.206900	-1.340300	1.835400
C	3.333500	-1.972400	-0.216000
H	4.434600	-1.978900	-0.270900
H	2.713500	3.394700	0.354200
O	1.211800	3.993700	-2.724100
O	1.833200	1.416100	-3.697800
O	1.451400	-1.411600	-1.602500
H	1.161500	-1.119800	-2.478100
C	2.926400	-3.424200	0.198400
C	3.255700	-2.627800	-2.670000
H	4.350400	-2.647400	-2.760700
H	2.863300	-2.317200	-3.647000
C	2.745800	-4.010400	-2.288900
H	3.039200	-4.743000	-3.047700
H	1.650600	-3.996500	-2.260800
C	3.305200	-4.404900	-0.927600
H	2.968500	-5.410200	-0.645400
H	4.402300	-4.448000	-0.998500
C	1.428900	-3.557800	0.524000
H	1.210500	-4.595400	0.802200
H	1.147100	-2.921300	1.370000
H	0.798900	-3.278400	-0.322600
C	3.720900	-3.851900	1.443800
H	3.563100	-4.919800	1.629800
H	4.795500	-3.690400	1.303200
H	3.408100	-3.317800	2.345200
C	-0.016800	4.064300	-0.045500
H	-0.317700	3.759300	0.964800
C	-1.293600	4.266800	-0.866600
H	-1.880800	3.345700	-0.910900

H	-1.907300	5.040500	-0.397400
H	-1.061800	4.589800	-1.884500
C	0.795600	5.355300	0.078300
H	1.626500	5.250900	0.781300
H	1.190200	5.669500	-0.890600
H	0.149400	6.151900	0.456200
C	-1.098100	-0.886000	1.639300
C	-0.949900	-0.142400	0.465500
C	-0.104900	0.972000	0.488500
C	0.633600	1.287700	1.632200
C	0.497500	0.546900	2.809100
C	-0.390200	-0.526800	2.790900
C	-1.675400	-0.514400	-0.800600
H	-1.260700	0.070300	-1.624600
H	-1.488000	-1.569700	-1.032400
C	-2.067300	-2.040700	1.663100
H	-1.783000	-2.789700	0.910500
H	-1.994600	-2.534200	2.636700
C	-3.203900	-0.262200	-0.754400
C	-3.528000	-1.610500	1.428200
H	-3.718000	-0.690500	1.996600
H	-4.171800	-2.373800	1.873200
C	-3.954800	-1.423900	-0.039000
H	-3.643800	-2.333600	-0.578900
H	-0.524200	-1.123300	3.688300
O	1.564100	2.308600	1.600100
O	-0.005900	1.715000	-0.659500
O	-3.456900	0.998900	-0.134000
H	-3.016400	1.001000	0.727000
C	-5.510200	-1.366900	-0.182700
C	-3.688800	-0.108700	-2.197400
H	-3.375100	-0.997500	-2.761200
H	-3.171200	0.753600	-2.632500
C	-5.199600	0.050900	-2.294500
H	-5.499800	0.149500	-3.343000
H	-5.502800	0.973500	-1.787000
C	-5.882700	-1.155000	-1.662300
H	-6.973700	-1.068900	-1.739400
H	-5.597000	-2.055500	-2.226000
C	-6.144900	-0.262200	0.676100
H	-7.231100	-0.264800	0.529900
H	-5.957500	-0.437000	1.740400
H	-5.756600	0.725400	0.423700
C	-6.128300	-2.710600	0.237600
H	-7.187300	-2.729600	-0.042400
H	-5.633200	-3.548800	-0.265300
H	-6.078700	-2.879100	1.316500
C	1.305000	0.950200	4.027600

C	0.713900	2.221200	4.654300
H	-0.301300	2.025200	5.015300
H	1.321300	2.549100	5.503200
H	0.669300	3.034600	3.925200
C	1.425100	-0.160400	5.069000
H	0.457800	-0.373600	5.535300
H	1.804100	-1.087400	4.627800
H	2.111100	0.148800	5.862000
H	2.314200	1.196500	3.673000

M06-2X/6-31G(d,p) Free Energy = -2007.756356

Transition states.

Endo **Chanel**

Grandione TS1

C	-1.783300	-0.303500	-1.278900
C	-1.854500	-0.276900	0.124900
C	-2.739600	-1.268600	0.748700
C	-3.009400	-2.557400	-0.057200
C	-2.143800	-2.718100	-1.275800
C	-1.928500	-1.502500	-1.971600
C	-1.581400	0.958800	0.925200
H	-1.546600	0.668100	1.981100
H	-0.600600	1.372700	0.674000
C	-1.432400	0.964200	-2.003600
H	-0.459500	1.330400	-1.646200
H	-1.316300	0.742400	-3.067500
C	-2.652000	2.075700	0.756500
C	-2.505100	2.055600	-1.812500
H	-3.495100	1.589400	-1.857700
H	-2.434000	2.724800	-2.673400
C	-2.395100	2.901100	-0.531500
H	-1.348000	3.234600	-0.445900
H	-1.746400	-1.522800	-3.041000
O	-3.835200	-3.356700	0.308700
O	-3.277100	-1.122800	1.832200
O	-3.948700	1.495600	0.651600
H	-4.071700	0.923000	1.423000
C	-3.236700	4.214000	-0.637400
C	-2.577600	2.963300	2.004900
H	-1.541000	3.303500	2.132700
H	-2.819100	2.344000	2.878200
C	-3.506800	4.165500	1.911300
H	-3.431000	4.772200	2.819300
H	-4.543600	3.817200	1.842800
C	-3.142100	4.996000	0.687100
H	-3.781600	5.884300	0.615200
H	-2.110500	5.359400	0.805500
C	-4.710400	3.949000	-0.981800
H	-5.253000	4.900300	-1.018300
H	-4.805900	3.480200	-1.966100
H	-5.190000	3.296400	-0.251400
C	-2.646900	5.122500	-1.728500
H	-3.127700	6.105800	-1.684700
H	-1.570800	5.268600	-1.582800
H	-2.806800	4.730300	-2.736300
C	-2.251200	-4.025200	-2.021900
H	-2.273500	-4.809400	-1.257200
C	-1.048600	-4.248500	-2.938100

H	-0.113600	-4.094300	-2.396800
H	-1.073300	-5.266300	-3.335900
H	-1.069000	-3.561500	-3.790200
C	-3.568900	-4.072000	-2.806000
H	-4.430800	-3.939000	-2.148400
H	-3.589600	-3.291400	-3.573100
H	-3.662500	-5.039400	-3.306800
C	1.601100	-0.107500	1.424000
C	1.503700	-0.152600	0.065300
C	0.756700	-1.242200	-0.543800
C	-0.130300	-2.053400	0.346500
C	-0.021700	-1.937900	1.794500
C	0.841000	-1.001700	2.272900
C	2.276200	0.762100	-0.840700
H	1.866100	0.651600	-1.851000
H	2.130000	1.808600	-0.544500
C	2.603600	0.820700	2.058400
H	2.393200	1.860300	1.773200
H	2.506900	0.767100	3.146100
C	3.792000	0.457400	-0.910900
C	4.048400	0.454100	1.655600
H	4.141100	-0.637000	1.634000
H	4.708400	0.812900	2.449300
C	4.544000	1.032300	0.318400
H	4.294900	2.106200	0.314900
H	0.991800	-0.912100	3.345200
O	-0.741400	-3.050300	-0.196600
O	0.813600	-1.526200	-1.744600
O	3.976400	-0.957800	-0.931900
H	3.348500	-1.317300	-1.576300
C	6.101800	0.972200	0.204300
C	4.333900	1.062500	-2.212600
H	4.085400	2.132500	-2.235200
H	3.805100	0.593900	-3.052200
C	5.839000	0.878800	-2.344800
H	6.193600	1.313400	-3.285100
H	6.068300	-0.192100	-2.378300
C	6.540300	1.534700	-1.161800
H	7.629100	1.431200	-1.249200
H	6.324100	2.613200	-1.180400
C	6.652500	-0.450500	0.385800
H	7.743700	-0.435700	0.285100
H	6.419600	-0.834400	1.384100
H	6.238800	-1.145500	-0.345900
C	6.741300	1.871000	1.275400
H	7.815800	1.962100	1.082500
H	6.310500	2.878400	1.255700
H	6.628700	1.468600	2.285500

C	-0.755400	-2.939500	2.660700
H	-1.685800	-3.207800	2.148400
C	-1.117000	-2.384300	4.036500
H	-1.679600	-1.450800	3.946000
H	-1.731100	-3.108900	4.577900
H	-0.223200	-2.197100	4.640000
C	0.081900	-4.222100	2.777900
H	0.301600	-4.637800	1.790800
H	1.029300	-4.010000	3.283300
H	-0.458100	-4.975900	3.358000

M06-2X/6-31G(d,p) Free Energy= -2007.690395

Grandione TS2

C	-1.710800	-0.378400	-1.315800
C	-1.874900	-0.367400	0.082900
C	-2.754700	-1.421500	0.623300
C	-2.896100	-2.684100	-0.250300
C	-1.687800	-2.861100	-1.165600
C	-1.542100	-1.600600	-1.942300
C	-1.726200	0.864700	0.913700
H	-1.732800	0.554300	1.964600
H	-0.756200	1.333400	0.721500
C	-1.466600	0.916300	-2.034500
H	-0.521600	1.346400	-1.668400
H	-1.319300	0.702700	-3.096800
C	-2.849400	1.921300	0.703300
C	-2.609700	1.932900	-1.859000
H	-3.566800	1.405900	-1.932100
H	-2.562300	2.615500	-2.710900
C	-2.586400	2.771100	-0.568200
H	-1.561500	3.156100	-0.441800
H	-1.415500	-1.657400	-3.017700
O	-3.808900	-3.455800	-0.114200
O	-3.365100	-1.336300	1.671600
O	-4.104000	1.268300	0.537800
H	-4.236200	0.693600	1.305900
C	-3.492100	4.039900	-0.693700
C	-2.877700	2.797800	1.961900
H	-1.868000	3.193800	2.136200
H	-3.119900	2.156300	2.818800
C	-3.867400	3.947300	1.840000
H	-3.863000	4.546400	2.756100
H	-4.879600	3.544200	1.723800
C	-3.495800	4.810300	0.640700
H	-4.178900	5.663700	0.548900
H	-2.491200	5.226600	0.806800
C	-4.933700	3.698900	-1.101800

H	-5.528200	4.618300	-1.145400
H	-4.963700	3.243200	-2.096600
H	-5.403300	3.006600	-0.401800
C	-2.907800	4.992300	-1.749800
H	-3.448100	5.944800	-1.721700
H	-1.850400	5.200300	-1.552700
H	-2.997700	4.601500	-2.766700
C	-1.688200	-4.149100	-1.979100
H	-1.868300	-4.952800	-1.254800
C	-0.331000	-4.375300	-2.645800
H	0.477300	-4.349600	-1.913400
H	-0.326800	-5.345700	-3.149200
H	-0.129600	-3.603600	-3.395500
C	-2.819700	-4.164000	-3.009900
H	-3.794200	-3.998800	-2.546600
H	-2.665300	-3.403600	-3.781800
H	-2.839700	-5.136000	-3.509700
C	1.580000	0.014800	1.478200
C	1.480600	-0.091000	0.110300
C	0.755500	-1.203300	-0.439000
C	-0.055200	-1.994900	0.462500
C	0.042900	-1.869800	1.877700
C	0.861700	-0.866300	2.342300
C	2.226900	0.809700	-0.830800
H	1.830900	0.634900	-1.837800
H	2.038000	1.862900	-0.587200
C	2.538900	1.016800	2.069300
H	2.278500	2.032700	1.742700
H	2.441400	1.003900	3.158500
C	3.756600	0.567100	-0.873600
C	4.001900	0.710100	1.688500
H	4.153700	-0.373700	1.725600
H	4.638700	1.145400	2.463100
C	4.474500	1.242200	0.324200
H	4.174700	2.301300	0.263900
H	0.985700	-0.734700	3.413700
O	-0.667100	-3.061700	-0.086500
O	0.706700	-1.497400	-1.665400
O	4.009200	-0.835800	-0.809400
H	3.424100	-1.263200	-1.451500
C	6.034700	1.250300	0.218700
C	4.281100	1.121000	-2.205200
H	3.989100	2.177000	-2.287100
H	3.776500	0.587000	-3.020300
C	5.793200	0.992800	-2.321800
H	6.135100	1.386300	-3.284500
H	6.066700	-0.067800	-2.291800
C	6.458700	1.746500	-1.177100

H	7.551400	1.683700	-1.253100
H	6.198100	2.811900	-1.262300
C	6.654900	-0.128500	0.493100
H	7.745100	-0.062100	0.402300
H	6.429900	-0.462900	1.510700
H	6.288600	-0.888300	-0.198400
C	6.622000	2.245400	1.233800
H	7.692900	2.374200	1.042100
H	6.145000	3.227900	1.145400
H	6.518800	1.904900	2.267300
C	-0.677000	-2.858500	2.771500
H	-1.566700	-3.202900	2.232300
C	-1.137900	-2.243100	4.091700
H	-1.755100	-1.357100	3.917500
H	-1.730100	-2.971300	4.652600
H	-0.288400	-1.957600	4.719800
C	0.217000	-4.083500	3.010000
H	0.512300	-4.544100	2.063200
H	1.124400	-3.789700	3.547500
H	-0.311400	-4.831300	3.608600

M06-2X/6-31G(d,p) Free Energy= -2007.691407

Isograndiona TS1

C	-2.179700	1.147100	-0.630200
C	-1.040300	0.326400	-0.659200
C	-0.172500	0.553600	-1.813100
C	-0.208000	1.954300	-2.454900
C	-0.916800	2.986400	-1.633900
C	-2.125900	2.457900	-1.110700
C	-0.974500	-1.010800	0.012100
H	0.070000	-1.339400	-0.013100
H	-1.241500	-0.912600	1.069800
C	-3.409800	0.636300	0.063900
H	-3.164600	0.426100	1.115500
H	-4.167400	1.424300	0.067200
C	-1.870300	-2.104200	-0.630200
C	-3.977100	-0.636400	-0.597400
H	-3.896600	-0.537900	-1.685000
H	-5.043400	-0.668100	-0.361500
C	-3.341500	-1.967800	-0.160500
H	-3.287500	-1.963800	0.940400
H	-2.977600	3.108200	-0.953800
O	0.284400	2.143700	-3.539800
O	0.534900	-0.292800	-2.343700
O	-1.858400	-1.972500	-2.049000
H	-0.932600	-1.952000	-2.331900
C	-4.250500	-3.186000	-0.524100

C	-1.280600	-3.462800	-0.229400
H	-1.188100	-3.496400	0.864900
H	-0.262000	-3.523600	-0.635200
C	-2.137700	-4.625900	-0.708600
H	-1.688100	-5.576600	-0.404700
H	-2.174900	-4.622200	-1.803700
C	-3.540900	-4.494900	-0.128700
H	-4.169400	-5.339800	-0.436000
H	-3.471100	-4.536800	0.968500
C	-4.622600	-3.222600	-2.014200
H	-5.244500	-4.102600	-2.213600
H	-5.205500	-2.339300	-2.293900
H	-3.741200	-3.259300	-2.655300
C	-5.552400	-3.125300	0.291600
H	-6.110800	-4.058100	0.157900
H	-5.344500	-3.008500	1.361000
H	-6.208900	-2.309200	-0.021500
C	-0.826900	4.428100	-2.091700
H	-1.275400	4.425000	-3.095400
C	0.615200	4.933400	-2.225500
H	1.243900	4.243300	-2.789900
H	0.605700	5.893900	-2.746900
H	1.058100	5.083500	-1.239100
C	-1.627900	5.360600	-1.180200
H	-2.706100	5.207200	-1.265400
H	-1.336300	5.194700	-0.139300
H	-1.421200	6.400400	-1.446000
C	1.448400	0.544900	2.114700
C	1.451300	1.139400	0.884900
C	0.320100	2.004700	0.557400
C	-0.782100	2.213800	1.553800
C	-0.845200	1.312500	2.701000
C	0.262200	0.572500	2.954400
C	2.633700	1.114700	-0.047200
H	2.320400	1.573500	-0.992100
H	3.413700	1.781300	0.348200
C	2.700200	-0.039700	2.727300
H	3.409600	0.788000	2.878300
H	2.446500	-0.401300	3.728000
C	3.309700	-0.251700	-0.380900
C	3.420200	-1.166600	1.980100
H	2.692800	-1.924700	1.677600
H	4.090000	-1.639700	2.703900
C	4.242300	-0.736600	0.758300
H	4.850400	0.137000	1.042200
H	0.299500	-0.029700	3.860000
O	-1.591800	3.120900	1.361700
O	0.394800	2.888100	-0.376500

O	2.360300	-1.292600	-0.567600
H	1.816400	-1.090400	-1.343900
C	5.263200	-1.848600	0.347300
C	4.075900	-0.009000	-1.692300
H	4.702800	0.884700	-1.570400
H	3.336200	0.225900	-2.469700
C	4.949800	-1.185000	-2.100200
H	5.478900	-0.952600	-3.030100
H	4.323000	-2.061500	-2.300200
C	5.942200	-1.479900	-0.983500
H	6.621500	-2.293100	-1.267400
H	6.566500	-0.587600	-0.826100
C	4.607100	-3.233300	0.224800
H	5.347900	-3.959900	-0.127800
H	4.242000	-3.581300	1.195900
H	3.762800	-3.222700	-0.466000
C	6.375600	-1.936900	1.403900
H	7.142800	-2.645300	1.072500
H	6.857200	-0.963900	1.549900
H	6.008600	-2.286200	2.372700
C	-2.042600	1.416800	3.616900
C	-2.367600	0.099000	4.319100
H	-1.580400	-0.181100	5.026400
H	-3.297200	0.196900	4.886100
H	-2.487000	-0.719300	3.601500
C	-1.831900	2.547300	4.634600
H	-0.983600	2.313300	5.286100
H	-1.628400	3.493900	4.127500
H	-2.721900	2.671900	5.258600
H	-2.894700	1.700800	2.987200

M06-2X/6-31G(d,p) Free Energy= -2007.681199

Isograndione TS2

C	-2.223100	1.145000	-0.627200
C	-1.049800	0.350000	-0.633300
C	-0.247900	0.554800	-1.854700
C	-0.419800	1.926300	-2.526300
C	-0.772400	2.972200	-1.477300
C	-2.090400	2.476900	-0.973000
C	-0.958100	-0.978700	0.048200
H	0.093300	-1.285100	0.028200
H	-1.229300	-0.868100	1.103300
C	-3.468700	0.615000	0.017400
H	-3.261800	0.458900	1.088200
H	-4.244600	1.383600	-0.040100
C	-1.831200	-2.102000	-0.578900
C	-3.979000	-0.694600	-0.609300

H	-3.882900	-0.627400	-1.697800
H	-5.047800	-0.756700	-0.391000
C	-3.308600	-1.990300	-0.120000
H	-3.262000	-1.946700	0.980300
H	-2.950000	3.136300	-1.019500
O	-0.262900	2.095200	-3.707300
O	0.490800	-0.267500	-2.373100
O	-1.820700	-1.998100	-1.999800
H	-0.897500	-2.018300	-2.289800
C	-4.183600	-3.244200	-0.447400
C	-1.208700	-3.436800	-0.147200
H	-1.116100	-3.441300	0.947400
H	-0.188100	-3.481800	-0.550200
C	-2.036000	-4.632700	-0.596900
H	-1.561000	-5.563300	-0.270600
H	-2.074900	-4.657400	-1.691700
C	-3.440600	-4.522500	-0.016800
H	-4.047700	-5.391400	-0.299000
H	-3.367500	-4.532000	1.080900
C	-4.556100	-3.334000	-1.935200
H	-5.152000	-4.237300	-2.108200
H	-5.164400	-2.476400	-2.239400
H	-3.674400	-3.363900	-2.576400
C	-5.485600	-3.195000	0.369200
H	-6.020700	-4.144500	0.260200
H	-5.278500	-3.046400	1.434700
H	-6.162900	-2.403600	0.037600
C	-0.757800	4.414100	-1.974700
H	-1.471100	4.438000	-2.809100
C	0.622500	4.824400	-2.497500
H	0.982500	4.150200	-3.276600
H	0.565900	5.832200	-2.916900
H	1.349300	4.837500	-1.681200
C	-1.217300	5.385900	-0.886500
H	-2.222400	5.164500	-0.521000
H	-0.535800	5.339500	-0.033100
H	-1.212500	6.404800	-1.282400
C	1.459800	0.622700	2.121200
C	1.459300	1.188100	0.864100
C	0.297800	1.943600	0.491100
C	-0.775700	2.200900	1.437000
C	-0.834200	1.397300	2.632000
C	0.292500	0.690800	2.951700
C	2.646200	1.134800	-0.063700
H	2.344200	1.584700	-1.016000
H	3.436300	1.790200	0.329500
C	2.715800	0.040800	2.727100
H	3.446900	0.856300	2.833900

H	2.476600	-0.281700	3.744800
C	3.300100	-0.246300	-0.382300
C	3.392300	-1.124500	1.997800
H	2.637800	-1.863500	1.713300
H	4.052300	-1.608400	2.723500
C	4.219200	-0.740600	0.763500
H	4.852900	0.120100	1.030700
H	0.336500	0.168900	3.905600
O	-1.636500	3.062900	1.125400
O	0.365700	2.815700	-0.541800
O	2.329700	-1.268000	-0.569900
H	1.789300	-1.054300	-1.345800
C	5.208900	-1.885200	0.366800
C	4.079600	-0.030000	-1.690500
H	4.728400	0.848300	-1.570400
H	3.351400	0.218100	-2.474400
C	4.924500	-1.230800	-2.087100
H	5.464000	-1.017500	-3.015600
H	4.275200	-2.091000	-2.285700
C	5.903200	-1.546900	-0.964400
H	6.558500	-2.382500	-1.239800
H	6.553800	-0.672900	-0.810800
C	4.515300	-3.252100	0.254000
H	5.240600	-4.004300	-0.076700
H	4.126000	-3.575200	1.224400
H	3.682100	-3.228100	-0.449900
C	6.315600	-1.995800	1.427700
H	7.069800	-2.719000	1.098200
H	6.815800	-1.032500	1.576000
H	5.938800	-2.338900	2.394800
C	-2.033300	1.549100	3.540400
C	-2.379200	0.256000	4.279100
H	-1.598200	-0.015300	4.996600
H	-3.309800	0.381500	4.839300
H	-2.506700	-0.580300	3.583900
C	-1.813400	2.705800	4.525200
H	-0.966600	2.485200	5.183500
H	-1.600800	3.635200	3.990100
H	-2.701500	2.858500	5.146000
H	-2.878600	1.824500	2.897700

M06-2X/6-31G(d,p) Free Energy= -2007.686878

Grandione β TS

C	-0.160700	-2.533400	-0.275600
C	0.194900	-2.172100	0.989400
C	-0.837700	-1.582600	1.843800
C	-2.314900	-1.569500	1.333200

C	-2.652700	-2.338100	0.091000
C	-1.538600	-2.516500	-0.748900
C	1.518900	-2.486600	1.637900
H	1.449900	-2.136400	2.672800
H	1.629100	-3.578600	1.686100
C	0.843600	-3.106900	-1.240800
H	1.072900	-4.137900	-0.931900
H	0.370600	-3.178300	-2.223600
C	2.824200	-1.911100	1.047700
C	2.148700	-2.311800	-1.377200
H	1.911400	-1.242100	-1.415400
H	2.573300	-2.568500	-2.350800
C	3.204300	-2.579200	-0.293700
H	3.192700	-3.660200	-0.080900
H	-1.708900	-3.035000	-1.684300
O	-3.110300	-0.891300	1.942300
O	-0.634900	-1.088800	2.936800
O	2.666700	-0.510000	0.804900
H	2.628000	-0.055600	1.659200
C	4.651600	-2.276100	-0.797000
C	3.921200	-2.120000	2.100900
H	3.951300	-3.185100	2.364600
H	3.633800	-1.579400	3.012200
C	5.292000	-1.677200	1.608900
H	6.040100	-1.839300	2.391100
H	5.278100	-0.601400	1.398500
C	5.655900	-2.463500	0.355700
H	6.655000	-2.186800	-0.002600
H	5.697400	-3.531800	0.614000
C	4.787700	-0.862200	-1.377500
H	5.816700	-0.701500	-1.719200
H	4.129600	-0.726200	-2.242400
H	4.536300	-0.096100	-0.642600
C	5.037200	-3.285700	-1.889700
H	6.095700	-3.163200	-2.143600
H	4.890900	-4.315400	-1.545400
H	4.466800	-3.148500	-2.812400
C	-3.745700	-3.411000	0.175300
H	-3.311500	-4.173100	0.839400
C	-4.008800	-4.066700	-1.181700
H	-3.144600	-4.623900	-1.551100
H	-4.838100	-4.771600	-1.084000
H	-4.284700	-3.311000	-1.922300
C	-5.057500	-2.947000	0.812000
H	-4.898700	-2.457400	1.773700
H	-5.578500	-2.251700	0.150100
H	-5.695600	-3.820500	0.973100
C	-2.110200	2.625200	-0.751700

C	-1.335800	1.620500	-1.274400
C	-1.935800	0.312000	-1.414900
C	-3.288200	0.088800	-0.886700
C	-4.005100	1.136200	-0.211800
C	-3.420100	2.370500	-0.215600
C	0.055100	1.854800	-1.793400
H	0.405300	0.913200	-2.230500
H	0.040700	2.592800	-2.607100
C	-1.574600	4.030600	-0.670000
H	-1.298900	4.381900	-1.674100
H	-2.370800	4.691600	-0.316500
C	1.100900	2.297200	-0.731800
C	-0.358300	4.158700	0.261900
H	-0.518800	3.533900	1.146900
H	-0.319000	5.194700	0.608500
C	0.989200	3.804800	-0.380800
H	1.036700	4.325900	-1.351400
H	-3.945600	3.215700	0.220900
O	-3.715000	-1.108500	-0.946300
O	-1.362300	-0.691200	-1.922100
O	0.895800	1.553500	0.463500
H	1.201000	0.644500	0.304500
C	2.186700	4.372400	0.448900
C	2.483300	1.985200	-1.324300
H	2.551000	2.462200	-2.311800
H	2.550300	0.902600	-1.486000
C	3.633000	2.462800	-0.449700
H	4.588700	2.232300	-0.933300
H	3.620400	1.914300	0.499600
C	3.513700	3.961900	-0.213600
H	4.343800	4.326400	0.404500
H	3.593100	4.475800	-1.183300
C	2.169100	3.907900	1.912700
H	3.026300	4.338200	2.443300
H	1.261900	4.249600	2.421100
H	2.208800	2.821200	1.994700
C	2.146700	5.909800	0.437300
H	3.078900	6.303300	0.857200
H	2.049600	6.295500	-0.583700
H	1.327500	6.315700	1.036600
C	-5.368800	0.822100	0.358600
H	-5.251400	-0.119600	0.907400
C	-6.386700	0.586000	-0.766700
H	-6.055200	-0.211600	-1.436600
H	-7.357100	0.303300	-0.347000
H	-6.522200	1.499600	-1.355400
C	-5.873400	1.890200	1.325700
H	-5.146800	2.087000	2.119400

H	-6.078600	2.833000	0.807200
H	-6.807300	1.560400	1.788600

M06-2X/6-31G(d,p) Free Energy= -2007.653529

Isograndione β TS

C	2.133900	2.158100	-0.523600
C	1.623700	1.390000	-1.550800
C	0.309300	1.737500	-2.094000
C	-0.358100	3.003400	-1.521600
C	-0.044700	3.307800	-0.080100
C	1.335100	3.083100	0.202900
C	2.440900	0.432200	-2.369000
H	1.787600	0.056700	-3.163100
H	3.244400	0.991000	-2.868900
C	3.572100	1.990700	-0.107600
H	4.214700	2.310200	-0.940500
H	3.772100	2.669500	0.724900
C	3.068800	-0.788300	-1.660500
C	3.953600	0.558400	0.308700
H	3.128200	0.119600	0.877200
H	4.805200	0.640600	0.989100
C	4.322100	-0.392900	-0.840200
H	4.964800	0.158900	-1.545000
H	1.825000	3.696000	0.947300
O	-1.054600	3.688300	-2.230300
O	-0.239600	1.167500	-3.021200
O	2.130100	-1.360200	-0.757800
H	1.258800	-1.375100	-1.178100
C	5.185000	-1.596700	-0.347400
C	3.418800	-1.803300	-2.758700
H	4.050000	-1.308800	-3.509900
H	2.488800	-2.093900	-3.263000
C	4.142400	-3.021600	-2.204400
H	4.371400	-3.724300	-3.012000
H	3.483900	-3.543800	-1.501000
C	5.424200	-2.579600	-1.508800
H	5.978500	-3.445200	-1.125200
H	6.074700	-2.092800	-2.250600
C	4.534400	-2.330000	0.832800
H	5.169900	-3.165600	1.147800
H	4.418700	-1.660500	1.692000
H	3.545700	-2.714800	0.575000
C	6.566200	-1.093400	0.101000
H	7.211900	-1.948200	0.330100
H	7.048700	-0.510400	-0.691200
H	6.517900	-0.474400	1.001000
C	-0.746300	4.515300	0.526600

H	-0.446800	5.360700	-0.107100
C	-0.293300	4.773800	1.965900
H	0.740300	5.121000	2.030300
H	-0.926300	5.543800	2.414300
H	-0.387600	3.855100	2.551800
C	-2.277700	4.415300	0.483600
H	-2.647300	4.095500	-0.491300
H	-2.634900	3.713400	1.240900
H	-2.701700	5.397700	0.707400
C	-0.818000	-1.567200	0.525300
C	-1.148600	-0.326400	0.034500
C	-0.565700	0.805300	0.683000
C	0.499300	0.657300	1.683900
C	0.849900	-0.681700	2.127800
C	0.161200	-1.718100	1.569200
C	-2.239500	-0.126200	-0.981000
H	-2.208200	0.909800	-1.331700
H	-2.057600	-0.753300	-1.861600
C	-1.589700	-2.775900	0.065000
H	-1.530000	-2.875400	-1.027300
H	-1.129200	-3.673300	0.487400
C	-3.669000	-0.395100	-0.440900
C	-3.070500	-2.718100	0.493600
H	-3.126000	-2.318100	1.511600
H	-3.438200	-3.746700	0.536400
C	-4.002300	-1.910200	-0.425600
H	-3.813400	-2.245300	-1.458600
H	0.341200	-2.730200	1.922300
O	1.015200	1.709500	2.119700
O	-1.023700	2.003300	0.504700
O	-3.768500	0.090500	0.897000
H	-3.485500	1.016200	0.890500
C	-5.506900	-2.238400	-0.156800
C	-4.645800	0.375300	-1.340000
H	-4.470700	0.079200	-2.383300
H	-4.402500	1.443800	-1.269300
C	-6.099200	0.118900	-0.969200
H	-6.763100	0.684400	-1.630900
H	-6.281400	0.475900	0.050500
C	-6.393200	-1.372400	-1.072100
H	-7.445100	-1.579400	-0.839600
H	-6.232000	-1.689100	-2.113300
C	-5.905500	-2.026900	1.311700
H	-6.969300	-2.257400	1.438900
H	-5.343600	-2.697200	1.969800
H	-5.723400	-1.003700	1.642400
C	-5.794200	-3.703900	-0.522800
H	-6.875100	-3.880600	-0.504000

H	-5.432600	-3.938000	-1.530400
H	-5.340500	-4.409800	0.177700
C	1.823800	-0.819600	3.278000
H	2.727000	-0.255400	3.004800
C	2.211800	-2.267300	3.571800
H	2.572400	-2.786700	2.680900
H	3.002100	-2.292800	4.327000
H	1.356000	-2.823600	3.970000
C	1.246900	-0.176000	4.551000
H	1.014300	0.878300	4.393400
H	0.330900	-0.696800	4.848900
H	1.966100	-0.256600	5.371500

M06-2X/6-31G(d,p) Free Energy= -2007.670788l

***Exo* Channel**

Grandione TS

C	-2.094700	-0.022500	0.056100
C	-2.994200	0.920000	-0.308900
C	-2.883500	2.279000	0.238500
C	-1.768700	2.552100	1.267300
C	-0.624600	1.599000	1.293400
C	-0.878500	0.297400	0.812800
C	-4.178900	0.620000	-1.181500
H	-4.636600	1.576200	-1.454000
H	-3.851300	0.145100	-2.114000
C	-2.296600	-1.458300	-0.342000
H	-2.316900	-1.531700	-1.437800
H	-1.435700	-2.044400	-0.010700
C	-5.267400	-0.264000	-0.519400
C	-3.590300	-2.056900	0.244100
H	-3.707900	-1.710100	1.276000
H	-3.450900	-3.139800	0.288600
C	-4.877500	-1.764700	-0.544900
H	-4.666500	-1.978300	-1.605200
H	-0.382800	-0.533900	1.306000
O	-1.850600	3.513300	1.997000
O	-3.658300	3.181200	-0.016600
O	-5.426600	0.120700	0.844800
H	-5.715500	1.043700	0.857000
C	-6.031200	-2.742300	-0.149100
C	-6.577200	-0.026300	-1.282500
H	-6.400200	-0.219000	-2.349200
H	-6.838700	1.036200	-1.193400
C	-7.708800	-0.912900	-0.782000
H	-8.623800	-0.709800	-1.347600
H	-7.921100	-0.676000	0.266700
C	-7.309400	-2.375600	-0.926900

H	-8.121000	-3.036100	-0.597500
H	-7.142000	-2.588400	-1.993100
C	-6.319700	-2.739400	1.359500
H	-7.138000	-3.435400	1.576200
H	-5.445200	-3.074600	1.926200
H	-6.593400	-1.747400	1.720900
C	-5.662500	-4.177000	-0.561700
H	-6.534600	-4.828300	-0.438300
H	-5.357100	-4.220600	-1.613100
H	-4.858100	-4.598100	0.047300
C	0.421800	1.857700	2.356500
H	0.611400	2.936900	2.358900
C	1.746600	1.108800	2.168000
H	2.362600	1.528200	1.369100
H	2.325700	1.183500	3.092800
H	1.590600	0.043700	1.968100
C	-0.184900	1.479200	3.721100
H	-1.133700	1.989000	3.897000
H	-0.351400	0.398400	3.776200
H	0.509800	1.755800	4.519100
C	3.621400	1.104400	-1.316700
C	2.595000	0.197400	-1.194600
C	1.273500	0.708300	-0.945600
C	1.074400	2.133300	-0.747000
C	2.190000	3.041500	-0.776200
C	3.407600	2.508900	-1.099700
C	2.788000	-1.283300	-1.384300
H	1.801600	-1.752500	-1.340900
H	3.201000	-1.487000	-2.380800
C	5.012400	0.625300	-1.645300
H	4.999100	0.088000	-2.603700
H	5.661400	1.494300	-1.782700
C	3.682900	-1.972400	-0.321700
C	5.623600	-0.286800	-0.566500
H	5.418100	0.146300	0.421500
H	6.709300	-0.242700	-0.683600
C	5.199000	-1.764800	-0.611300
H	5.337300	-2.105000	-1.650500
H	4.273500	3.159700	-1.184500
O	-0.103900	2.489200	-0.405600
O	0.249000	-0.034300	-0.797100
O	3.323500	-1.497800	0.975400
H	3.512500	-0.550100	1.007800
C	6.149600	-2.658800	0.250200
C	3.321600	-3.459900	-0.322200
H	3.419300	-3.836200	-1.349100
H	2.265200	-3.544100	-0.043400
C	4.202100	-4.281300	0.609000

H	3.911200	-5.335900	0.564400
H	4.051100	-3.951400	1.642700
C	5.661900	-4.119100	0.206700
H	6.312300	-4.724800	0.849600
H	5.788500	-4.499500	-0.817800
C	6.244900	-2.185000	1.709000
H	6.892200	-2.865500	2.273600
H	6.690200	-1.186800	1.769900
H	5.267300	-2.148000	2.191400
C	7.565700	-2.648500	-0.348100
H	8.179400	-3.405900	0.151800
H	7.545300	-2.885700	-1.417600
H	8.071800	-1.688200	-0.218600
C	1.923900	4.508000	-0.521600
H	1.256300	4.555100	0.349200
C	1.171100	5.126500	-1.709500
H	0.235900	4.595600	-1.902800
H	0.937900	6.175600	-1.505500
H	1.789300	5.082100	-2.612000
C	3.189400	5.303900	-0.213800
H	3.756900	4.857500	0.607700
H	3.842200	5.359600	-1.091300
H	2.926100	6.327300	0.065000

M06-2X/6-31G(d,p) Free Energy= -2007.664611

Isograndione TS

C	2.467500	0.393000	0.224900
C	2.521800	-0.931400	-0.047200
C	1.718500	-1.868700	0.748700
C	0.876400	-1.295000	1.906900
C	0.578800	0.164700	1.867300
C	1.477800	0.971900	1.139500
C	3.455900	-1.514600	-1.068400
H	3.175100	-2.562800	-1.210600
H	3.322200	-1.013100	-2.034200
C	3.424900	1.342500	-0.440300
H	3.286700	1.296900	-1.529100
H	3.178500	2.364500	-0.141400
C	4.955600	-1.466700	-0.676700
C	4.893300	1.035500	-0.086400
H	4.955500	0.772900	0.974800
H	5.456600	1.963000	-0.215800
C	5.566500	-0.063200	-0.925600
H	5.345200	0.151800	-1.983700
H	1.693200	1.966000	1.520900
O	0.481900	-2.031800	2.781000
O	1.730100	-3.075400	0.595100

O	5.090600	-1.759500	0.712900
H	4.743300	-2.650200	0.860100
C	7.124000	-0.003600	-0.810300
C	5.683000	-2.533800	-1.505000
H	5.473600	-2.353800	-2.568000
H	5.251500	-3.513000	-1.259900
C	7.186700	-2.524000	-1.268300
H	7.668000	-3.300500	-1.871400
H	7.391300	-2.760500	-0.218000
C	7.748000	-1.153400	-1.623800
H	8.836000	-1.129900	-1.485000
H	7.562300	-0.965700	-2.691700
C	7.613900	-0.066600	0.644300
H	8.709100	-0.033000	0.662700
H	7.250200	0.792100	1.217600
H	7.278600	-0.973200	1.148900
C	7.641700	1.304700	-1.431200
H	8.734000	1.266800	-1.505100
H	7.241500	1.449100	-2.440900
H	7.389800	2.184400	-0.833200
C	-0.192300	0.712900	3.050500
H	-1.011800	0.010600	3.243800
C	-0.768800	2.123500	2.870800
H	-1.630500	2.155800	2.204100
H	-1.103400	2.487500	3.846200
H	-0.017600	2.831600	2.504600
C	0.745200	0.703600	4.273400
H	1.181200	-0.282200	4.443400
H	1.554700	1.427500	4.134100
H	0.182900	0.992700	5.165600
C	-3.569200	1.778200	0.116900
C	-2.862300	0.642200	0.424900
C	-1.453400	0.643300	0.162300
C	-0.779600	1.854200	-0.271800
C	-1.533600	3.068400	-0.439500
C	-2.892400	2.974400	-0.297200
C	-3.510200	-0.604300	0.956100
H	-2.713000	-1.276100	1.293600
H	-4.118000	-0.365000	1.837500
C	-5.074400	1.760700	0.185300
H	-5.402000	1.553300	1.213800
H	-5.453000	2.755300	-0.067100
C	-4.388400	-1.373400	-0.064000
C	-5.700900	0.723000	-0.767400
H	-5.143300	0.724700	-1.709700
H	-6.714100	1.062700	-0.998300
C	-5.784000	-0.716000	-0.229000
H	-6.189000	-0.660400	0.794600

H	-3.508800	3.850300	-0.483800
O	0.488600	1.804100	-0.379300
O	-0.684100	-0.344200	0.429300
O	-3.753000	-1.363500	-1.340900
H	-2.869200	-1.740800	-1.230200
C	-6.812900	-1.576000	-1.032700
C	-4.517900	-2.818100	0.436000
H	-4.913900	-2.799900	1.460400
H	-3.511800	-3.253500	0.493000
C	-5.426600	-3.654200	-0.453200
H	-5.489300	-4.678900	-0.073100
H	-4.996800	-3.709300	-1.459700
C	-6.811500	-3.021900	-0.498800
H	-7.494000	-3.621800	-1.113400
H	-7.226800	-3.017500	0.519900
C	-6.530600	-1.577400	-2.542900
H	-7.271200	-2.204500	-3.052200
H	-6.613700	-0.568300	-2.958700
H	-5.532100	-1.952300	-2.770900
C	-8.233100	-1.030200	-0.812300
H	-8.963000	-1.725900	-1.240300
H	-8.454500	-0.926500	0.255700
H	-8.392700	-0.060600	-1.291300
C	-0.784400	4.321900	-0.829100
H	0.115600	4.345100	-0.199700
C	-1.583300	5.600500	-0.589800
H	-1.956000	5.657400	0.436900
H	-0.952500	6.473500	-0.776400
H	-2.440200	5.664200	-1.269000
C	-0.320300	4.239000	-2.290900
H	0.294800	3.351400	-2.457800
H	-1.186700	4.192100	-2.958700
H	0.267800	5.122800	-2.555100

M06-2X/6-31G(d,p) Free Energy= -2007.663921

Grandione β TS

C	2.290400	0.536300	-1.809400
C	3.360900	0.819300	-1.042700
C	4.103300	-0.282800	-0.415800
C	3.876600	-1.697300	-0.969700
C	2.560100	-1.965400	-1.573800
C	1.847300	-0.853000	-2.083500
C	3.745600	2.223100	-0.671900
H	4.743400	2.191800	-0.222300
H	3.813000	2.851400	-1.567100
C	1.381100	1.647000	-2.260700
H	1.951600	2.393600	-2.827700

H	0.618000	1.244400	-2.931200
C	2.778800	2.878900	0.347800
C	0.693300	2.322300	-1.053500
H	0.440900	1.545800	-0.324500
H	-0.244700	2.755900	-1.414100
C	1.498100	3.422200	-0.342700
H	1.873200	4.113400	-1.115100
H	1.347600	-0.995100	-3.038600
O	4.769200	-2.518900	-0.897900
O	4.886100	-0.124900	0.503100
O	2.386100	1.900500	1.309300
H	3.191800	1.465500	1.624900
C	0.586700	4.292200	0.582900
C	3.535800	4.012500	1.050600
H	3.923500	4.704300	0.290500
H	4.405600	3.578300	1.559700
C	2.649200	4.766000	2.032600
H	3.218400	5.562700	2.522200
H	2.317500	4.078000	2.818200
C	1.447900	5.350600	1.299000
H	0.808000	5.913400	1.989800
H	1.810500	6.069700	0.549500
C	-0.183600	3.454000	1.615500
H	-0.788600	4.117100	2.244200
H	-0.869500	2.755400	1.124000
H	0.485600	2.874700	2.252700
C	-0.441100	5.056800	-0.267600
H	-0.951800	5.798900	0.355400
H	0.043300	5.588400	-1.094100
H	-1.211400	4.401700	-0.684700
C	2.301800	-3.382000	-2.038400
H	3.192900	-3.640600	-2.628800
C	1.069600	-3.528000	-2.933800
H	1.170200	-2.974100	-3.870800
H	0.930700	-4.581600	-3.188800
H	0.160200	-3.187900	-2.425800
C	2.253600	-4.394700	-0.879700
H	3.078400	-4.233800	-0.183200
H	1.309300	-4.336800	-0.331600
H	2.335700	-5.405400	-1.287400
C	-2.034000	-2.639700	0.657000
C	-1.592400	-1.912400	-0.421100
C	-0.234800	-1.479900	-0.407100
C	0.696000	-1.987500	0.583100
C	0.234500	-2.925400	1.579400
C	-1.110800	-3.168800	1.621800
C	-2.520600	-1.356000	-1.459300
H	-1.922900	-1.057300	-2.327100

H	-3.224100	-2.123200	-1.799700
C	-3.516900	-2.755000	0.890100
H	-4.010600	-3.229900	0.032200
H	-3.698800	-3.398000	1.755700
C	-3.313400	-0.120200	-0.965400
C	-4.146300	-1.365200	1.147400
H	-3.459600	-0.782800	1.771100
H	-5.051800	-1.524600	1.738500
C	-4.529800	-0.543900	-0.100100
H	-5.111200	-1.211200	-0.757300
H	-1.522000	-3.786100	2.416400
O	1.902600	-1.622300	0.458300
O	0.210500	-0.644700	-1.273400
O	-2.457200	0.700900	-0.168300
H	-1.656400	0.873500	-0.686000
C	-5.501000	0.629000	0.250700
C	-3.770900	0.671300	-2.196600
H	-4.344800	0.003300	-2.853300
H	-2.877600	0.978900	-2.756100
C	-4.618500	1.876700	-1.813900
H	-4.926000	2.425100	-2.710100
H	-4.013600	2.561000	-1.208100
C	-5.842600	1.413500	-1.031800
H	-6.475900	2.267300	-0.760900
H	-6.448500	0.766300	-1.683200
C	-4.930900	1.584400	1.310000
H	-5.667600	2.366500	1.526100
H	-4.728600	1.056400	2.247200
H	-4.003300	2.055000	0.981900
C	-6.828000	0.064900	0.786500
H	-7.569800	0.868700	0.845000
H	-7.225300	-0.711100	0.122700
H	-6.731300	-0.357500	1.790000
C	1.245100	-3.454900	2.569500
H	2.143100	-3.702300	1.988300
C	0.771700	-4.710500	3.297200
H	0.452100	-5.485500	2.594400
H	1.582200	-5.113300	3.910200
H	-0.066300	-4.487900	3.966000
C	1.635500	-2.358500	3.572600
H	2.018600	-1.473600	3.058200
H	0.764600	-2.065000	4.167800
H	2.408500	-2.725100	4.254500

M06-2X/6-31G(d,p) Free Energy= -2007.658750

Isograndione β TS

C	-2.714700	-0.830900	1.540500

C	-3.047200	-1.875400	0.753100
C	-1.964800	-2.719000	0.244500
C	-0.684600	-2.759700	1.090400
C	-0.261800	-1.473400	1.710400
C	-1.320000	-0.562500	1.945100
C	-4.442700	-2.155100	0.279800
H	-4.445300	-3.149000	-0.180700
H	-5.127900	-2.194400	1.134700
C	-3.751200	0.197900	1.902300
H	-4.558700	-0.279900	2.474300
H	-3.298300	0.949100	2.555000
C	-4.980800	-1.144700	-0.760000
C	-4.342300	0.903800	0.662300
H	-3.527800	1.118600	-0.034000
H	-4.734200	1.864900	1.005400
C	-5.468200	0.159900	-0.075400
H	-6.193800	-0.180600	0.681500
H	-1.218400	0.085800	2.811600
O	-0.133400	-3.828400	1.235400
O	-2.055700	-3.421400	-0.746200
O	-3.933000	-0.811600	-1.669700
H	-3.525700	-1.643000	-1.954600
C	-6.273000	1.116800	-1.014400
C	-6.124200	-1.832000	-1.518800
H	-6.858100	-2.201800	-0.789800
H	-5.712100	-2.712000	-2.028800
C	-6.807100	-0.897000	-2.505600
H	-7.615400	-1.421500	-3.025300
H	-6.085300	-0.583700	-3.268200
C	-7.354800	0.314400	-1.761900
H	-7.878000	0.989700	-2.450200
H	-8.101100	-0.031000	-1.030900
C	-5.371800	1.844800	-2.023100
H	-5.987200	2.470000	-2.680000
H	-4.664000	2.505000	-1.511500
H	-4.797300	1.146800	-2.633200
C	-7.008000	2.175200	-0.176200
H	-7.702200	2.731400	-0.815400
H	-7.590200	1.709900	0.627000
H	-6.327500	2.904300	0.271300
C	0.974300	-1.426100	2.597000
H	0.624100	-1.785600	3.575500
C	1.533700	-0.006200	2.799600
H	0.762200	0.755800	2.942400
H	2.163400	-0.003300	3.692800
H	2.165400	0.292900	1.958300
C	2.106500	-2.352900	2.139200
H	1.878300	-3.403500	2.298700

H	2.282000	-2.212900	1.066100
H	3.015800	-2.109600	2.699500
C	2.474800	1.876200	-0.785600
C	2.127800	0.548600	-0.732500
C	0.813300	0.226400	-0.237600
C	-0.040900	1.274800	0.300300
C	0.441600	2.624600	0.409000
C	1.645400	2.884500	-0.184900
C	2.995400	-0.541300	-1.297300
H	2.468000	-1.490700	-1.155000
H	3.098900	-0.412500	-2.383700
C	3.731400	2.315900	-1.494200
H	3.672500	2.005200	-2.547400
H	3.762200	3.409100	-1.497400
C	4.422000	-0.680600	-0.690600
C	5.040900	1.786500	-0.890300
H	4.989600	1.865400	0.200000
H	5.840000	2.456800	-1.218500
C	5.416200	0.350800	-1.284200
H	5.305000	0.263600	-2.377000
H	2.010100	3.907200	-0.227900
O	-1.193200	0.922900	0.716400
O	0.335400	-0.955800	-0.182700
O	4.391400	-0.452100	0.717000
H	3.973300	-1.213700	1.136600
C	6.923700	0.055100	-0.992500
C	4.873400	-2.120200	-0.977000
H	4.767600	-2.306500	-2.054200
H	4.177300	-2.805400	-0.473800
C	6.309900	-2.389100	-0.553300
H	6.579500	-3.425000	-0.782500
H	6.405900	-2.266700	0.531900
C	7.234800	-1.424600	-1.283600
H	8.283900	-1.619500	-1.029600
H	7.134700	-1.597800	-2.365400
C	7.325300	0.395000	0.451200
H	8.376000	0.128000	0.611000
H	7.228100	1.468100	0.643700
H	6.710300	-0.132600	1.181200
C	7.804600	0.884600	-1.940900
H	8.851600	0.583200	-1.826100
H	7.521300	0.721400	-2.986500
H	7.753100	1.957000	-1.734700
C	-0.442600	3.660400	1.069700
H	-1.449200	3.531200	0.651400
C	0.008800	5.095300	0.810200
H	0.082900	5.309900	-0.259600
H	-0.707300	5.794000	1.250100

H	0.984900	5.289500	1.267600
C	-0.533700	3.391600	2.578900
H	-0.937800	2.395200	2.776500
H	0.457200	3.465600	3.039600
H	-1.189900	4.124600	3.057300

M06-2X/6-31G(d,p) Free Energy= -2007.654042

Intermediates in the grandione and isograndione Domino Channel.

Grandione INT

C	1.875400	0.508200	-1.381100
C	1.711100	0.545800	0.125400
C	2.779600	1.487700	0.681600
C	2.878100	2.821900	-0.096400
C	1.721100	2.899200	-1.080100
C	1.884800	1.708100	-1.978300
C	1.720800	-0.796100	0.850800
H	1.673400	-0.569700	1.920700
H	0.808200	-1.351400	0.618100
C	1.734400	-0.782800	-2.130700
H	0.765000	-1.230000	-1.858200
H	1.668000	-0.534300	-3.194200
C	2.940300	-1.742200	0.621100
C	2.845600	-1.819300	-1.925000
H	3.814400	-1.314000	-1.974200
H	2.801200	-2.502900	-2.777200
C	2.766300	-2.643000	-0.632500
H	1.750600	-3.062600	-0.556100
H	1.905400	1.822500	-3.057400
O	3.710000	3.653400	0.160100
O	3.442100	1.299700	1.673800
O	4.145000	-1.012800	0.411400
H	4.320300	-0.502600	1.213500
C	3.729600	-3.874800	-0.698300
C	3.053100	-2.576800	1.907100
H	2.072800	-3.026400	2.115800
H	3.265300	-1.887400	2.735200
C	4.103700	-3.671400	1.819500
H	4.139600	-4.231200	2.759500
H	5.093500	-3.223700	1.675000
C	3.761400	-4.597400	0.660300
H	4.473900	-5.428800	0.597800
H	2.772300	-5.041300	0.846800
C	5.159800	-3.486600	-1.107800
H	5.805700	-4.370500	-1.059700
H	5.188400	-3.118000	-2.137600
H	5.572500	-2.710400	-0.461800
C	3.196900	-4.887300	-1.725300
H	3.809900	-5.794700	-1.698800
H	2.164000	-5.172700	-1.497800
H	3.229500	-4.505100	-2.749100
C	1.539600	4.247400	-1.767300
H	1.488700	4.990100	-0.961000
C	0.229600	4.279400	-2.555700
H	-0.618200	4.008000	-1.925600

H	0.067600	5.280100	-2.965100
H	0.265100	3.576700	-3.395100
C	2.730000	4.585600	-2.668000
H	3.672700	4.571100	-2.118800
H	2.802800	3.885000	-3.506200
H	2.593400	5.584800	-3.089700
C	-1.679400	-0.287100	1.475300
C	-1.542700	-0.320900	0.127900
C	-0.768200	0.754500	-0.497200
C	0.345000	1.422700	0.328200
C	-0.013000	1.518300	1.797200
C	-0.931800	0.670500	2.291200
C	-2.377700	-1.194400	-0.763400
H	-1.945500	-1.163100	-1.770100
H	-2.332600	-2.236100	-0.425700
C	-2.753300	-1.111600	2.131700
H	-2.624100	-2.172600	1.881800
H	-2.664700	-1.027000	3.218000
C	-3.859100	-0.754600	-0.869800
C	-4.158200	-0.641400	1.692600
H	-4.160800	0.452400	1.636400
H	-4.856200	-0.920500	2.486000
C	-4.678100	-1.220400	0.364600
H	-4.525700	-2.311600	0.402900
H	-1.179100	0.707200	3.349400
O	0.584100	2.718800	-0.178500
O	-1.003900	1.138100	-1.634000
O	-3.917200	0.668600	-0.939200
H	-3.206300	0.953900	-1.533700
C	-6.222700	-1.027000	0.218700
C	-4.432600	-1.353000	-2.161500
H	-4.279000	-2.440800	-2.147300
H	-3.851900	-0.958500	-3.004900
C	-5.913800	-1.044400	-2.327500
H	-6.290100	-1.480300	-3.258600
H	-6.049700	0.040200	-2.402600
C	-6.686800	-1.596000	-1.136200
H	-7.761200	-1.403000	-1.246100
H	-6.563600	-2.689100	-1.114900
C	-6.648900	0.443700	0.342900
H	-7.735400	0.521200	0.220500
H	-6.401900	0.839600	1.333000
H	-6.162900	1.075400	-0.401700
C	-6.956800	-1.829700	1.305800
H	-8.030600	-1.840400	1.089500
H	-6.609300	-2.868500	1.332900
H	-6.833900	-1.399700	2.303200
C	0.641500	2.585300	2.656100

H	1.468100	3.031600	2.096600
C	1.203600	2.026700	3.965300
H	1.935900	1.237100	3.773800
H	1.696100	2.824600	4.528200
H	0.409200	1.616900	4.596300
C	-0.367300	3.713900	2.915900
H	-0.739600	4.124300	1.973600
H	-1.220100	3.337000	3.489000
H	0.104200	4.520000	3.485600

M06-2X/6-31G(d,p) Free Energy= -2007.712221

Isograndione INT

C	-2.168100	1.294000	-0.848000
C	-0.885800	0.536600	-0.542700
C	-0.122900	0.497600	-1.866700
C	-0.082600	1.861900	-2.590100
C	-0.562400	2.927900	-1.624300
C	-1.988300	2.503300	-1.400500
C	-1.026700	-0.839400	0.105300
H	-0.012700	-1.241600	0.193900
H	-1.385400	-0.715700	1.131900
C	-3.484900	0.829600	-0.302800
H	-3.388700	0.769200	0.793200
H	-4.214400	1.620700	-0.501200
C	-1.920600	-1.913600	-0.584000
C	-4.025100	-0.503300	-0.832800
H	-3.893300	-0.539300	-1.917900
H	-5.102000	-0.503700	-0.644600
C	-3.418400	-1.765400	-0.205400
H	-3.436900	-1.645200	0.889800
H	-2.808400	3.178700	-1.621700
O	0.287700	1.977600	-3.729900
O	0.466900	-0.447400	-2.343300
O	-1.868000	-1.821200	-2.003800
H	-0.954600	-1.974800	-2.279100
C	-4.305900	-3.018800	-0.507200
C	-1.351100	-3.267000	-0.126900
H	-1.284800	-3.262300	0.969600
H	-0.321200	-3.343500	-0.501000
C	-2.192400	-4.450500	-0.578000
H	-1.741700	-5.385000	-0.229100
H	-2.213200	-4.492300	-1.672900
C	-3.601200	-4.298100	-0.022000
H	-4.223500	-5.162000	-0.285600
H	-3.542800	-4.274300	1.076400
C	-4.646700	-3.153100	-2.000100
H	-5.206700	-4.080200	-2.165700

H	-5.278000	-2.324300	-2.334800
H	-3.751000	-3.164000	-2.622600
C	-5.623200	-2.915100	0.277800
H	-6.199800	-3.837700	0.149600
H	-5.433900	-2.783800	1.348800
H	-6.254000	-2.089200	-0.061900
C	-0.354400	4.367300	-2.078700
H	-0.867700	4.451100	-3.045400
C	1.131500	4.678000	-2.277200
H	1.600000	3.984500	-2.979100
H	1.249400	5.691500	-2.669000
H	1.660500	4.617300	-1.322300
C	-0.977500	5.345500	-1.081200
H	-2.057100	5.207600	-0.981900
H	-0.528700	5.203800	-0.094600
H	-0.796400	6.373700	-1.405300
C	1.415700	0.578500	2.123500
C	1.296700	0.990900	0.845700
C	-0.024300	1.581100	0.361800
C	-0.922000	2.043200	1.531800
C	-0.878600	1.271600	2.786700
C	0.270000	0.615300	3.040500
C	2.463900	0.961600	-0.109600
H	2.150400	1.378500	-1.071700
H	3.230100	1.660400	0.256500
C	2.740600	0.142300	2.709400
H	3.430500	0.998400	2.673100
H	2.586900	-0.074800	3.770400
C	3.169400	-0.406100	-0.379800
C	3.421500	-1.067500	2.058400
H	2.678400	-1.851100	1.883300
H	4.136900	-1.463400	2.784700
C	4.165000	-0.769700	0.749700
H	4.775500	0.134400	0.905700
H	0.400500	0.115900	3.999000
O	-1.642000	3.016700	1.405100
O	0.255300	2.711200	-0.441700
O	2.236200	-1.479500	-0.446200
H	1.688600	-1.359900	-1.235200
C	5.181400	-1.903600	0.395300
C	3.877200	-0.252100	-1.734900
H	4.499300	0.652400	-1.701500
H	3.107900	-0.079800	-2.499900
C	4.744800	-1.452500	-2.084300
H	5.227000	-1.297200	-3.054700
H	4.117300	-2.346300	-2.177200
C	5.791300	-1.648000	-0.995100
H	6.461900	-2.480300	-1.242100

H	6.416000	-0.743900	-0.944700
C	4.543300	-3.300900	0.433500
H	5.285500	-4.051200	0.138000
H	4.208500	-3.550600	1.445200
H	3.681200	-3.373800	-0.230900
C	6.344100	-1.880900	1.399800
H	7.113000	-2.597000	1.089000
H	6.807300	-0.889100	1.443700
H	6.033000	-2.158000	2.410500
C	-2.022200	1.443900	3.760200
C	-2.258000	0.195000	4.608600
H	-1.418200	0.009100	5.286000
H	-3.152200	0.324700	5.224400
H	-2.394900	-0.691900	3.982500
C	-1.790000	2.676900	4.645000
H	-0.897500	2.532100	5.262700
H	-1.647600	3.570800	4.033300
H	-2.644900	2.840100	5.308100
H	-2.921200	1.634500	3.160200

M06-2X/6-31G(d,p) Free Energy= -2007.710883
